# Theoretical Reactivity Study of Indol-4-Ones and Their Correlation with Antifungal Activity

**DOI:** 10.3390/molecules22030427

**Published:** 2017-03-08

**Authors:** María de los Ángeles Zermeño-Macías, Marco Martín González-Chávez, Francisco Méndez, Rodolfo González-Chávez, Arlette Richaud

**Affiliations:** 1 Posgrado en Ciencias Farmacobiológicas, Universidad Autónoma de San Luis Potosí, 78210 San Luis Potosí, Mexico; angeles.zermeno@uaslp.mx; 2 Facultad de Ciencias Químicas, Universidad Autónoma de San Luis Potosí, Av. Dr. Manuel Nava No. 6 Zona Universitaria, 78210 San Luis Potosí, Mexico; rogochau@gmail.com; 3 Departamento de Química, División de Ciencias Básicas e Ingeniería, Universidad Autónoma Metropolitana, Unidad Iztapalapa, 09340 Ciudad de México, Mexico; arletterichaud@gmail.com

**Keywords:** DFT, HSAB principle, indol-4-ones, Fukui function, MEP, antifungal activity, structure-activity analysis

## Abstract

Chemical reactivity descriptors of indol-4-ones obtained via density functional theory (DFT) and hard–soft acid–base (HSAB) principle were calculated to prove their contribution in antifungal activity. Simple linear regression was made for global and local reactivity indexes. Results with global descriptors showed a strong relationship between antifungal activity vs. softness (*S*) (r = 0.98) for series I (**6**, **7a**–**g**), and for series II (**8a**–**g**) vs. chemical potential (*µ*), electronegativity (*χ*) and electrophilicity (*ω*) (r = 0.86), *p* < 0.05. Condensed reactivity descriptors *s*_k_^+^, *ω*_k_^−^ for different fragments had strong relationships for series I and II (r = 0.98 and r = 0.92). Multiple linear regression was statistically significant for *S* (r = 0.98), *η* (r = 0.91), and *µ*/*ω* (r = 0.91) in series I. Molecular electrostatic potential maps (MEP) showed negative charge accumulation around oxygen of carbonyl group and positive accumulation around nitrogen. Fukui function isosurfaces showed that carbons around nitrogen are susceptible to electrophilic attack, whereas the carbon atoms of the carbonyl and phenyl groups are susceptible to nucleophilic attack for both series. The above suggest that global softness in conjunction with softness and electrophilicity of molecular fragments in enaminone systems and pyrrole rings contribute to antifungal activity of indol-4-ones.

## 1. Introduction

Azole antifungal compounds have been used as therapeutic options for the treatment of systemic fungal infections. Mostly triazoles (fluconazole, etc.) and imidazoles (ketoconazole, etc.) [1,2] are used effectively against yeast and filamentous fungi. However, some etiologic agents have developed resistance by different mechanisms; moreover, azole compounds also present toxicity or side effects [2,3,4,5]. Consequently, some studies to discover new antifungal agents have been done [3,6].

**Figure 1:**
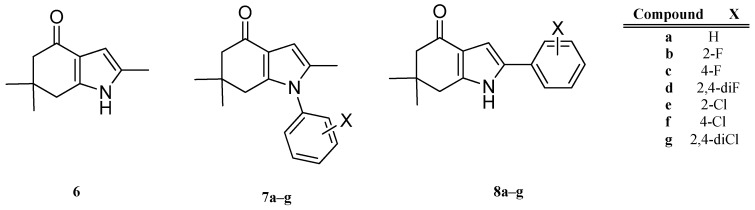
Indol-4-ones **6**, **7a**–**g** and **8a**–**g** designed, synthetized and tested by Gonzalez et al. [6].

Recently, González et al. designed and synthesized a series of novel indol-4-one derivatives with 1- and 2-(2,4-substituted phenyl) side chains (Figure 1). These compounds were tested in vitro against eight human pathogenic filamentous fungus and yeast strains by determination of the minimal inhibitory concentration (MIC); as MIC decreased, the antifungal activity increased. Based on their results, they reported activity against *Candida tropicalis*, *Candida guilliermondii* and *Candida parapsilosis* at MIC values of 0.0316 mM (8 µg·mL^−1^) for compounds **8a**–**g**; and MIC values of 0.1014 mM (31.25 µg·mL^−1^) against *Aspergillus fumigatus* (Table 1) for compounds **7a**–**g**. A change in the position of the halophenyl regioisomers from N-1 to C-2 increased the antifungal activity. It was the first report about antifungal activity for these indol-4-one derivatives.

Density functional theory (DFT) and the hard–soft acid–base principle (HSAB) have been used to study the biological activity of some biomolecules. Fukui functions were used for understanding the reactivity of nitrogenous bases of DNA and RNA [7,8]; chemical hardness was used to study the dopamine drug–receptor interactions [9]; the relationship between different biological activity and chemical reactivity indices, such as electrophilicity, hardness, and electronegativity was used for testosterone derivatives and their biological activity [10,11]; the dipolar moment, ionization potential, electronic affinity, electronegativity, electrophilicity, and others showed the inhibitory activity of carbonic anhydrase [12]; quantum chemical descriptors were used to study protoporphyrinogen oxidase inhibitors [13,14]; Fukui functions, softness, and electrostatic potential were useful for an antituberculotic drug design [15]; hardness, electronegativity, softness and electrophilicity has been applied to study the toxicity in specific species [16]; different descriptors were used to study mosquito repellent [17]; parameters such as dipolar moment were used to study chemical radioactivity protector [18]; ionization potential and charge were used to study antioxidants [19]; other activities and chemical reactivity parameters have been used in the study of nonnucleoside HIV-1 reverse transcriptase inhibitors [20], histone deacetylase inhibitors [21], and anti-HIV-1 integrase [22]; anti-HIV activity vs. electronegativity, hardness, chemical power, and electrophilicity was evaluated [23,24]; and others [25,26]. Within the above lies the importance of understanding the biological activity in a particular molecular set [27] and therefore for rational drug design.

In this work we made a DFT-HSAB reactivity study of the indol-4-one derivatives **6**–**8** to understand which molecular fragments are essential for antifungal activity. The development of new antifungal drugs can be based on obtaining good relationships between DFT-HSAB reactivity descriptors and antifungal activity. Based on the biological activity results reported by González et al. [6], we classified the indol-4-one derivatives **6**–**8** into two series according to the structure and biological activity. Series I includes compounds **6** and **7a**–**g**, while series II includes compounds **8a**–**g**.

## 2. Theoretical Methods

The geometries of the molecules **6**, **7a**–**g** and **8a**–**g** (Figure 1) were fully optimized at the B3LYP/6-311+G (d,p) level of theory using the Gaussian 09 program package [28]. For all stationary points, vibrational analyses were carried out. The ionization potential *I* = E_*N*−1_ − E*_N_* and the electronic affinity *A* = E*_N_* − E_*N* + 1_ were calculated at the geometry of the neutral species using the respective vertical energies E*_N_*, E_*N* + 1_, and E_*N* − 1_ of the systems with *N*, *N* + 1 and *N* − 1 electrons. The global reactivity indexes, chemical potential $\mu =-\frac{1}{2}\left(I+A\right)$, electronegativity χ=−µ, hardness $\eta =\frac{1}{2}\left(I-A\right)$, softness S=1/η and electrophilicity $\omega =\frac{\chi^{2}}{2\eta }$ [29,30,31], were calculated.

The local Fukui functions for nucleophilic $f^{+}\left(r\right)$, electrophilic $f^{-}\left(r\right)$, and radical $f^{0}\left(r\right)$ attacks were calculated using Equations (1)–(3) [32].

(1) $$f^{+}\left(r\right)=\rho_{N+1}\left(r\right)-\rho_{N}\left(r\right)\;\mathrm{Nucleophilic}\;\mathrm{attack}$$

(2) $$f^{-}\left(r\right)=\rho_{N}\left(r\right)-\rho_{N-1}\left(r\right)\;\mathrm{Electrophilic}\;\mathrm{attack}$$

(3) $$f^{0}\left(r\right)=\frac{1}{2}\left[\rho_{N+1}\left(r\right)-\rho_{N-1}\left(r\right)\right]\;\mathrm{Radical}\;\mathrm{attack}$$

where $\rho_{N+1}\left(r\right)$, $\rho_{N}\left(r\right)$ and $\rho_{N-1}\left(r\right)$ are the electronic densities for the systems with *N* + 1, *N* and *N* − 1 electrons, respectively, calculated with the geometry of the neutral species.

The condensed Fukui functions were calculated using the charge of each atom $q_{k}$ instead of the electron density ρ (r) (Equations (4)–(6)) [32,33,34,35]. The Hirshfeld population analysis scheme was used for the systems with *N*, *N* − 1 and *N* + 1 number of electrons. The condensed softness $s_{k}^{+}=Sf_{k}^{+}$, $s_{k}^{o}=Sf_{k}^{o}$ and $s_{k}^{-}=Sf_{k}^{-}$ and condensed electrophilicity indexes $\omega_{k}^{+}=S\omega_{k}^{+}$, $\omega_{k}^{o}=\omega f_{k}^{o}$, $\omega_{k}^{-}=\omega f_{k}^{-}$ were obtained. The local Fukui function isosurfaces were plotted with GaussView 5.0 [36].

Condensed Fukui functions:

(4) $$f_{k}^{+}=q_{k}\left(N+1\right)-q_{k}\left(N\right)\;\mathrm{Nucleophilic}\;\mathrm{attack}$$

(5) $$f_{k}^{-}=q_{k}\left(N\right)-q_{k}\left(N-1\right)\;\mathrm{Electrophilic}\;\mathrm{attack}$$

(6) $$f_{k}^{0}=\frac{1}{2}(q_{k}\left(N+1\right)-q_{k}\left(N-1\right))\;\mathrm{Radical}\;\mathrm{attack}$$

where $q_{k}$ is the electronic population value of *k*^th^ atom in the molecule.

## 3. Structure-Activity Relationship (SAR) Statistical Procedure

A simple and multiple regression analysis were made for the antifungal activities and the global and condensed reactivity indexes for each series of compounds. The Pearson and Determination Coefficients were obtained using SAS software [37] considering *p* < 0.05 as a significant value; the analysis was made for each time of testing: 24 and 48 h for yeast; and 48 and 72 h for filamentous fungus.

## 4. Results and Discussion

### 4.1. Global Reactivity Parameters

Table 2 shows the values of the calculated global chemical reactivity parameters for the 15 indol-4-ones compounds. The chemical reactivity values vary with the molecular structure and the substituent. According to the structural homology, the analyzed compounds were divided into two series: series I that includes compounds **6** and **7a**–**g** (N-1 substitution with phenyl moieties) and series II that includes compounds **8a** to **8g** (C-2 substitution with phenyl moieties). Table 2 shows that for series I compound **6** has the highest hardness value (4.18 eV) and **7g** has the lowest hardness value (3.80 eV); the difference is 0.38 eV. In contrast, for series II the highest hardness value (3.84 eV) corresponds to compound **8c** and the lowest value (3.73 eV) to **8f** and the difference is 0.11 eV. According to the maximum hardness principle, compounds **7g** and **8f** (**8g** and **8d** also) are more reactive than **6** and **8c**, respectively. The electronegativity equalization principle assures in the course of a chemical reaction energetic stabilization through equalization of middle HOMO-LUMO levels among ligand and receptor active molecular structures [38]. Table 2 reflects that compounds **7g** in series I and **8g** in series II present the highest electronegativity values (3.90 eV and 3.87 eV, respectively). The electrophilicity index *ω* value for the same compounds (**7g** 2.00 eV and **8g** 2.01 eV), reflects the ability of **7g** and **8g** to behave as the stronger electrophiles on each series. The relative change between the maximum and minimum values of *ω* in the Series I of Table 2 (ω_max_ − ω_min_/ω_max_) = 0.21 is larger than the corresponding change of 0.17 for series II. This indicates that the capacity of series I to accept electrons (electrophilic character) is more sensitive to the specific substituent than series II.

Simple linear regression of the minimum inhibitory concentration (MIC) vs. global reactivity parameters for both series was obtained (Table 3 and Table 4). The Pearson coefficient was positive and the relationships were directly proportional: when the antifungal activity decreased, the global reactivity values increased. Then, when the global reactivity of those 15 indol-4-ones decreases, the higher antifungal activity is obtained. The best statistically significant relationships (the Pearson coefficient *p* < 0.05) between both variables were obtained for yeast in series I: global hardness for *C. glabrata* 48 h (r*_η_* = 0.98), *C. krusei* 24 h (r*_η_* = 0.95), *C. tropicalis* 24 h (r*_η_* = 0.95), *C. guilliermondii* 24 (r*_η_* = 0.96) and 48 h (r*_η_* = 0.94), and fungi: *A. fumigatus* 72 h (r*_η_* = 0.79) (Table 3. This means a strong linear relationship between hardness and biological activity (96%, r^2^ values until 0.96), with only 4% of variance of activity left to explain after taking into account the hardness in a linear way. For series II, global electronegativity and global electrophilicity index had a higher Pearson coefficient for *C. albicans* 48 h and *C. glabrata* 24 h (r*_χ,ω_* = 0.98) and *C. tropicalis* 48 h (r*_χ_* = 0.82 and r*_ω_* = 0.80) (Table 4). This shows the same tendency as series I, with electronegativity and electrophilicity.

The relationship was strong for almost all cases, except for *C. parapsilosis* where the relationship did not have statistical significance.

Pearson coefficient in simple linear regression for series I had the following hierarchy from higher to lower values: *η* > *ω* > *χ* while *χ* = *ω* > *η* for series II. This could be related to results obtained by Putz et al. [24], where they report values of monolinear correlation of activity of uracil derivatives (anti-HIV action) vs. chemical reactivity indices, and the tendency shown was *η* > *ω* > *χ*, which is not the tendency one may expect obeying the established hierarchy for chemical binding scenario given by Putz [39], according which a chemical reaction/interaction is triggered by the electronegativity difference, followed by chemical hardness and electrophilicity: *χ* > *η* > *ω*, due to chemical–biological interactions. The higher Pearson coefficient presented by Putz is 0.67 for hardness, lower than the calculated value of the same parameter, 0.98. Although a different pharmacological activity is evaluated, it is possible to see the relation that can exist with these electronic properties of systems.

Stachowicz et al. evaluated thioamides derivatives and their activity against *C. albicans* and correlated their activity vs. hardness, softness, and electrophilicity, with r values around 0.72 to 0.93 [40]. These results coincide with those obtained by us with r values around 0.73 to 0.98; the chemical structure for thioamides are similar to indol-4-ones, =only in the presence of *N*-heterocyclic system of five members, and this similarity could be responsible for similar correlations between biological activities and chemical reactivity parameters.

Different biological activities have been correlated with chemical reactivity parameters: hardness, softness, chemical potential, electronegativity, electrophilicity, and other electronic parameters looking for any relationship between electronic parameters and biological activity. Examples of studies with different parameters are: for testosterone derivatives r*_ω_* = 0.42–0.94 [10,11]; carbonic anhydrase inhibitory r_*χ,µ*,S,εLUMO_ = 0.92 [12]; anti HIV-1 integrase r_LogP,*χ*_ = 0.93 [22]; anti-HIV activity with uracil derivatives r*_χ_* = 0.24, r*_η_* = 0.65, r*_ω_* = 0.65, r*_ω,χ_* = 0.69, and r_*ω,η*_ = 0.68, [24]; etc. Although our analysis of antifungal activity does not match with those described above, the obtained values of r are better.

Multiple lineal regression for global reactivity indexes indicated that both hardness and softness are significant variables for series I (see Table 5). The relationship was strong for *C. guilliermondii* 24 h (r = 0.99) and 48 h (r = 0.99). Hardness and electrophilicity as well as hardness and chemical potential had strong relationship for fungi, and are indicated specifically for *A. fumigatus* 48 h (r_*η,s*_ = 0.91) and 72 h (r*_η,µ_* = 0.91). For series II, there is no statistically significance (*p* > 0.05) linking two or more descriptors.

### 4.2. Local and Fragment Reactivity Parameters

**Figure 2:**
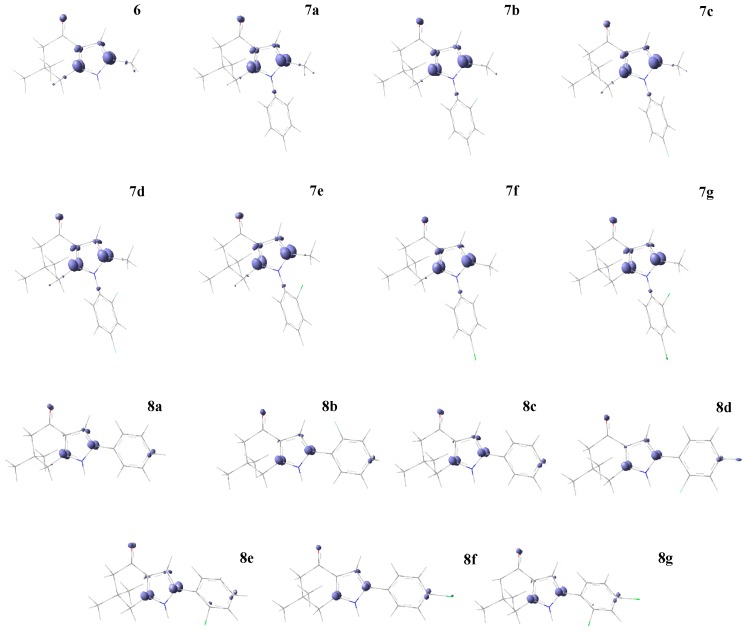
Fukui function isosurface plots for an electrophilic attack $f^{-}\left(r\right)$ of series I and II of compounds **6**, **7a**–**g**, and **8a**–**g**. In purple (positive values) are the favorable sites for an electrophilic attack; cutting value 0.01 a.u.

The local Fukui function is related with the frontier controlled soft–soft interactions. Figure 2 shows the isosurface plot of the Fukui function for an electrophilic attack $f^{-}\left(r\right)$, and the positive values are shown in purple. For series I, the carbon atoms neighboring the nitrogen atom of the pyrrole ring are susceptible to be attacked by a soft electrophile followed by the oxygen atom of the carbonyl group and the vinylic carbon atoms of the pirrolic ring. For compounds in series II, the Fukui function shows the same reactive sites than series I. In addition, the carbon atom in the *para*-position of the phenyl ring is susceptible for electrophilic attack.

**Figure 3:**
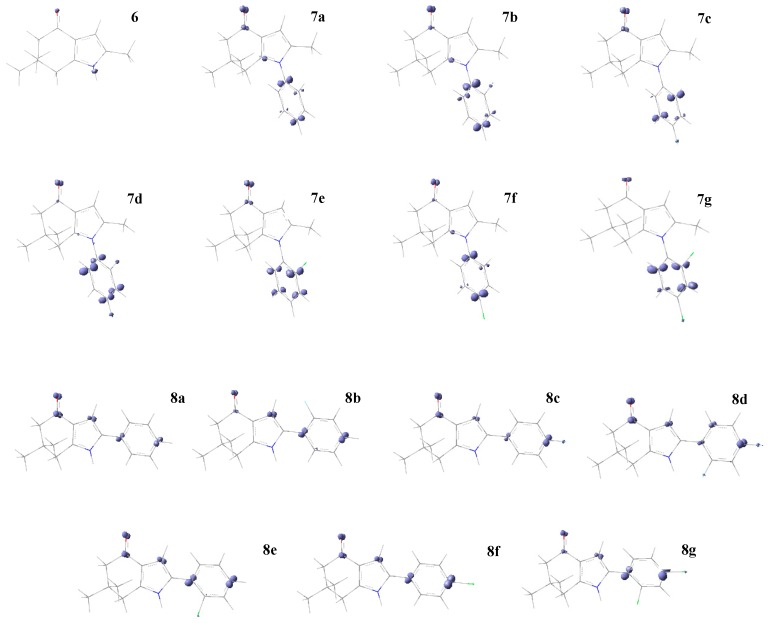
Fukui function isosurface plots for a nucleophilic attack $f^{+}\left(r\right)$ of series I (**6**, **7a**–**g**) and II (**8a**–**g**) of compounds. In purple color (positive values) are the favorable sites for a nucleophilic attack; cutting value 0.01 a.u.

Figure 3 shows the Fukui function for nucleophilic attack $f^{+}\left(r\right)$, and the regions in purple color are positive values and show the most favorable sites for the attack of a soft nucleophile. For series I, the carbonyl and phenyl carbon atoms are prone to nucleophilic attack. For series II, these regions are the carbonyl group and carbon atoms from vinyl and phenyl ring.

The local Fukui function is localized within the carbonyl, pyrrole and phenyl moieties. In order to understand which molecular fragments are responsible for antifungal activity, the softness and electrophilicity were calculated for different fragments of compounds **6**, **7a**–**g** and **8a**–**g**. Table 6 shows the ID of the analyzed fragment, microorganisms, experimental time of testing, fragment chemical reactivity parameter, statistical correlation coefficient (r) for MIC and softness and electrophilicity fragments, and the atoms (marked in orange) considered in the fragment for series I and II. The basic fragment ID a (g and i) is related with the oxygen atom, fragment ID b (f and h) includes the carbon atom to gets the carbonyl group, fragment ID c includes carbon and nitrogen atoms from pyrrole ring and *ipso*- and *ortho*-carbon atoms of the phenyl ring, fragment ID d includes *meta* and *para*-carbon atoms of the phenyl ring, and so on. For *Aspergillus niger* in series I, high correlation values were obtained for *s*_k_^−^ [fragment a = g (oxygen atom, r = 0.90, 48 h) and fragment b = f (carbonyl group, r = 0.93, 48 h)] and *s*_k_^+^ [fragment c (r = 0.98, 72 h) and fragment d (r = 0.95, 48 h)]. As we can observe for *Aspergillus niger*, the addition of the pyrrole and phenyl fragments to the carbonyl group increases the correlation coefficient for series I (the time of testing more representative for this species was 72 h). For *A. fumigatus*, linear regressions for *s*_k_^+^ (or *s*_k_^0^) includes the carbonyl group r = 0.83 (or r = 0.82) and the oxygen atom r = 0.81 (or r = 0.80). The more significant time of testing was 48 h.

For series II, *C. albicans* has the higher correlation values for *ω*_k_^−^; the addition of carbon atom to oxygen atom to obtain the carbonyl group keeps the correlation for *ω*_k_^−^ (see fragments m, r = 0.92 and n, r = 0.92). Fragments that include the nitrogen atom of the pyrrole ring do not increase the correlation value when increasing the number of carbon atoms of the phenyl ring (j, k and l, r = 0.75). The addition of the pyrrole ring, *ipso* carbon atom, and the *meta*-C atom from the phenyl ring, improves the correlation (q, r = 0.86). For other species, linear regressions with higher r were found when oxygen was included in *ω*_k_^0^: *C. krusei* 48 h (r = 0.78), *C. tropicalis* 24 h (r = 0.78), *C. guilliermondii* 48 h (r = 0.76), and *C. parapsilosis* 24 h (r = 0.78).

Additionally, the Parr functions [41,42] were calculated for electrophilic *P*^−^(r) and nucleophilic *P*^+^(r) attacks. They had similar tendency than Fukui functions (See Tables S1 and S2).

### 4.3. MEP and Dipole Moment

**Figure 4:**
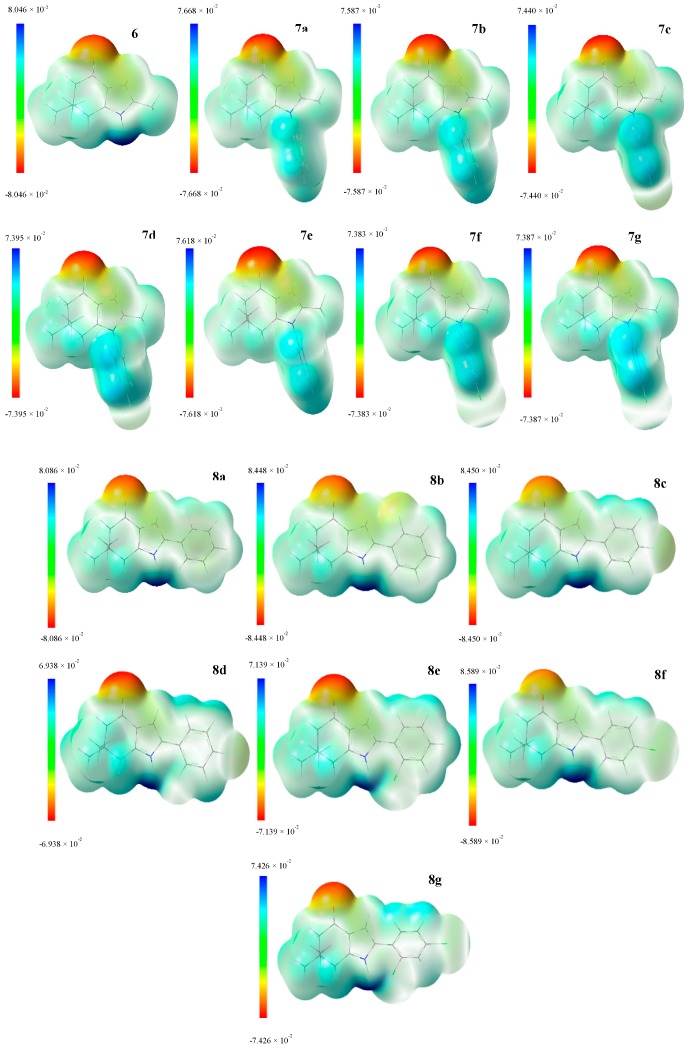
Molecular electrostatic potential maps from series I (**6**, **7a**–**g**) and II (**8a**–**g**). This chart shows regions with negative values (red), and positive values (blue). The color code is different range depending of the structure; units are given in a.u. for each scale.

Figure 4 shows the molecular electrostatic potential (MEP) for compounds **6**, **7a**–**g** and **8a**–**g**. The MEP is a useful descriptor for understanding which sites in the molecules have affinity to a proton (charge controlled hard-hard interactions [43]) and the relative polarity of the molecule [44,45,46,47,48,49,50,51,52]. Regions in red color indicate higher negative charge, higher electron density, and higher affinity to a proton. Regions in blue color indicate more positive charge, a low electron density and a low affinity to a proton. For series I and II, the red region is located near the oxygen atom from carbonyl group, and the blue region is located near the nitrogen atom for both series, and the phenyl group for series I. In general, compounds in series I show a higher dipolar moment than compounds of series II as we can observe in Table 7. The dipole moment was calculated for all of the 15 indol-4-ones at the B3LYP/6-311+G (d, p) level of theory. The dipole moment follows the trend: **7b** > **7e** > **7a** > **8b** > **6** > **7d** = **8a** > **7g** > **8f** > **8c** > **7c** > **7f** > **8e** > **8g** > **8d**. For series I, compound **7b** shows the highest value (7.26 D), and, for series II, compound **8b** has the highest value (6.69 D). Both compounds present a 2-fluor substitution in the aromatic ring. This can suggest that there is a correlation between the relative polarity of the compounds **6**, **7a**–**g** and **8a**–**g** and the kind of interactions that these compounds can have with the active site of the receptor to antifungal activity.

## 5. Conclusions

Hardness, electronegativity, and electrophilicity of indol-4-ones were the chemical reactivity parameters that had a higher correlation with antifungal activity. Hardness was the index that had higher correlation for series I, and chemical potential, electronegativity, and electrophilicity had higher correlation with antifungal activity for series II.

Fukui function for electrophilic attack had the higher correlation with molecular fragments around both pyrrole and carbonyl groups, suggesting that nature of the reactivity operative between the electrophilic sites of the indol-4-ones and the biologically active site in the studied fungi.

The strongest correlation with biological activity was found with *C. albicans*, *C. glabrata,* and *A. fumigatus*.

The molecular electrostatic potential and the dipole moment calculated for compounds **6**, **7a**–**g** and **8a**–**g** suggest that there is a correlation between the relative polarity of the compounds and the kind of interactions that these compounds can have with the active site of the receptor, as has been suggested by González et al.

## Figures and Tables

**Table 1:**
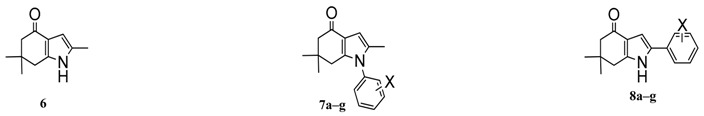
MIC in vitro of **6**, **7a**–**g** and **8a**–**g** against yeast and filamentous fungus.

Compound	X	*C. albicans*		*C. glabrata*		*C. krusei*		*C. tropicalis*		*C. guilliermondii*		*C. parapsilosis*		*A. niger*		*A. fumigatus*	
		24 h	48 h	24 h	48 h	24 h	48 h	24 h	48 h	24 h	48 h	24 h	48 h	48h h	72 h	48 h	72 h
**6**		1.4105	2.8210	2.8210	5.6420	2.8210	5.6420	1.4105	5.6420	1.4105	2.8210	0.0451	0.3526	0.7052	1.4105	0.7052	1.4105
**7a**	H	0.2467	1.9736	0.9868	3.9473	0.9868	3.9473	0.4934	3.9473	0.4934	0.4934	0.0316	0.2467	0.2467	0.4934	0.2467	0.4934
**7b**	2-F	0.2304	1.8428	0.4607	3.6856	0.9214	3.6856	0.4607	3.6856	0.4607	0.4607	0.0590	0.2304	0.2304	0.2304	0.1152	0.4607
**7c**	4-F	0.2304	1.8428	0.4607	3.6856	0.9214	3.6856	0.4607	3.6856	0.4607	0.4607	0.0590	0.9214	0.4607	0.9214	0.2304	0.9214
**7d**	2,4-diF	0.4321	0.8641	0.8641	3.4564	0.4321	0.8641	0.2160	0.4321	0.4321	0.4321	0.0553	0.2160	0.2160	0.4321	0.4321	0.8641
**7e**	2-Cl	0.4344	0.8687	0.8687	3.4748	0.4344	0.8687	0.2172	0.4344	0.4344	0.4344	0.1086	0.1086	0.2172	0.8687	0.1086	0.4344
**7f**	4-Cl	0.4344	0.8687	0.8687	3.4748	0.4344	0.8687	0.2172	0.4344	0.4344	0.4344	0.0556	0.2154	0.4344	>0.8687	0.4344	0.8687
**7g**	2,4-diCl	0.3879	0.7758	0.7758	3.1034	0.3879	0.7758	0.1940	0.3879	0.3879	0.3879	0.0970	0.7758	>0.7758	>0.7758	0.3879	0.3879
**8a**	H	0.5223	1.0447	0.5223	4.1787	0.5223	1.0445	0.2612	0.5223	0.2612	0.5223	0.0669	1.0447	>1.0447	>1.0447	1.0447	1.0447
**8b**	2-F	0.4858	0.9716	0.4858	0.9716	0.4858	0.9716	0.2429	0.4858	0.2429	0.2429	0.0622	0.2410	>0.9716	>0.9716	0.9716	0.9716
**8c**	4-F	0.2429	0.9716	0.4858	0.9716	0.2429	0.4858	0.1214	0.2429	0.1214	0.2429	0.0622	0.2410	>0.9716	>0.9716	>0.9716	>0.9716
**8d**	2,4-diF	0.2270	0.9081	0.4541	0.9081	0.2270	0.4541	0.1135	0.2270	0.1135	0.2270	0.0581	0.9081	>0.9081	>0.9081	>0.9081	>0.9081
**8e**	2-Cl	0.2283	0.9132	0.4566	0.9132	0.2283	0.4566	0.1142	0.2283	0.1142	0.1142	0.1142	0.9132	>0.9132	>0.9132	>0.9132	>0.9132
**8f**	4-Cl	0.2283	0.9132	0.4566	0.9132	0.2283	0.4566	0.1142	0.2283	0.1142	0.1142	0.0584	0.2265	>0.9132	>0.9132	>0.9132	>0.9132
**8g**	2,4-diCl	0.2028	0.8112	0.4056	0.8112	0.2028	0.4056	0.1014	0.1014	0.1014	0.1014	0.1014	0.2012	>0.8112	>0.8112	>0.8112	>0.8112

This table was modified and taken from González et al. paper [6], the MIC values were changed from µg·mL^−1^ to mM.

**Table 2:**
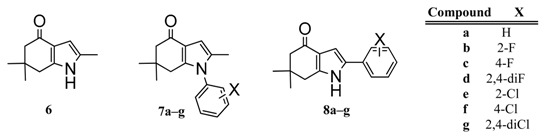
Global reactivity descriptors for the 15 compounds indol-4-ones **6**, **7a**–**g** and **8a**–**g**.

Compound	*η* (eV)	*χ* (eV)	*ω* (eV)
**6**	4.18	3.63	1.58
**7a**	3.92	3.65	1.70
**7b**	3.89	3.74	1.80
**7c**	3.91	3.77	1.82
**7d**	3.91	3.81	1.86
**7e**	3.89	3.71	1.76
**7f**	3.85	3.84	1.92
**7g**	3.80	3.90	2.00
**8a**	3.82	3.57	1.67
**8b**	3.80	3.67	1.77
**8c**	3.84	3.64	1.72
**8d**	3.74	3.77	1.90
**8e**	3.78	3.73	1.84
**8f**	3.73	3.72	1.85
**8g**	3.74	3.87	2.01

**Table 3:**
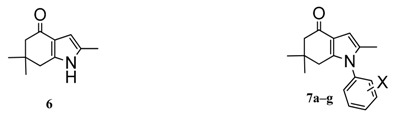
Pearson coefficient for each simple lineal regression for series I: Compounds **6** and **7a**–**g**.

Microorganism	Time of Testing (h)	*η* (eV)		*χ* (eV)		*ω* (eV)	
		r	*p*	r	*p*	r	*p*
*C. albicans*	24	0.87	0.0048	0.43	0.2868	0.60	0.1124
	48	0.82	0.0128	0.77	0.0246	0.83	0.0099
*C. glabrata*	24	0.90	0.0022	0.55	0.1602	0.70	0.0550
	48	0.98	0.00002	0.74	0.0358	0.86	0.0055
*C. krusei*	24	0.95	0.0003	0.69	0.0572	0.82	0.0126
	48	0.76	0.0283	0.75	0.0302	0.80	0.0166
*C. tropicalis*	24	0.95	0.0003	0.69	0.0572	0.82	0.0126
	48	0.74	0.0363	0.75	0.0331	0.79	0.0199
*C. guilliermondii*	24	0.96	0.0002	0.61	0.1109	0.76	0.0285
	48	0.94	0.0004	0.57	0.1393	0.73	0.0396
*C. parapsilosis*	24	0.46	0.2571	0.41	0.3145	0.46	0.2544
	48	0.15	0.7242	0.39	0.3419	0.33	0.4221
*A. niger*	48	0.75	0.0500	0.25	0.5875	0.47	0.2899
	72	0.78	0.0650	0.49	0.3236	0.68	0.1398
*A. fumigatus*	48	0.61	0.1062	0.01	0.9797	0.22	0.6061
	72	0.79	0.0191	0.29	0.4893	0.49	0.2188

h = hours, *η* = hardness, *χ* = electronegativity, and *ω* = electrophilicity index. In gray color is indicated the values that are statically significant *p* < 0.05.

**Table 4:**
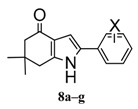
Pearson coefficient for simple lineal regression for series II: Compounds **8a**–**g**.

Microorganism	Time of Testing (h)	*η* (eV)		*χ* (eV)		*ω* (eV)	
		r	*p*	r	*p*	r	*p*
*C. albicans*	24	0.54	0.2155	0.71	0.0731	0.70	0.0821
	48	0.78	0.0406	0.98	0.00006	0.98	0.0002
*C. glabrata*	24	0.78	0.0406	0.98	0.00006	0.98	0.0002
	48	0.44	0.3173	0.66	0.1060	0.63	0.1285
*C. krusei*	24	0.54	0.2155	0.71	0.0731	0.70	0.0821
	48	0.54	0.2155	0.71	0.0731	0.70	0.0821
*C. tropicalis*	24	0.54	0.2155	0.71	0.0731	0.70	0.0821
	48	0.58	0.1673	0.82	0.0250	0.80	0.0315
*C. guilliermondii*	24	0.54	0.2155	0.71	0.0731	0.70	0.0821
	48	0.61	0.1488	0.78	0.0400	0.76	0.0472
*C. parapsilosis*	24	0.14	0.7712	0.49	0.2594	0.44	0.3164
	48	0.13	0.7774	0.27	0.5546	0.2547	0.5814

h = hours, *η* = hardness, *χ* = electronegativity, and *ω* = electrophilicity index. In gray color is indicated the values that are statically significant *p* < 0.05.

**Table 5:**
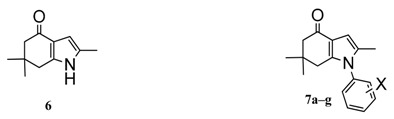
Multiple regression analysis for series I: Compounds **6** and **7a**–**g**.

Microorganism	Time of Testing (h)	r	r^2^	*p*	Significant Indexes
*C. albicans*	24	0.97	0.94	0.00070	*η*, *S*
	48	0.83	0.70	0.01000	*ω*
*C. glabrata*	24	0.97	0.94	0.00100	*η*, *S*
	48	0.98	0.96	<0.0001	*η*
*C. krusei*	24	0.95	0.91	0.00030	*η*
	48	0.80	0.64	0.01660	*ω*
*C. tropicalis*	24	0.95	0.91	0.00030	*η*
	48	0.79	0.62	0.02000	*ω*
*C. guilliermondii*	24	0.99	0.99	<0.0001	*η*, *S*
	48	0.99	0.99	<0.0001	*η*, *S*
*C. parapsilosis*	24	0.72	0.51	0.60230	No variable
	48	0.49	0.24	0.89800	No variable
*A. niger*	48	0.75	0.57	0.05070	*η*
	72	0.78	0.61	0.06480	*η*
*A. fumigatus*	48	0.91	0.84	0.01080	*η*, *ω*
	72	0.91	0.83	0.01140	*η*, *µ*

**Table 6:** Chemical reactivity criterions by fragment for series I (fragment ID: a–i) and series II (fragment ID: j–z).

ID	Microorganism	Time of Testing (h)	Fragment Chemical Parameter	r	Atoms Considered in the Fragment (Marked in Orange)
**a**	*Aspergillus fumigatus*	48	*s*_k_^+^	0.81	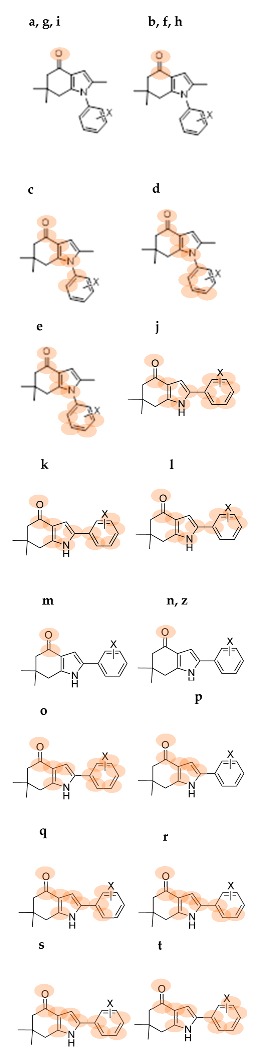
			*ω*_k_^+^	0.77	
**b**	*Aspergillus fumigatus*	48	*s*_k_^+^	0.83	
			*ω*_k_^+^	0.79	
**c**	*Aspergillus niger*	72	*s*_k_^+^	0.98	
			*ω*_k_^+^	0.89	
**d**	*Aspergillus niger*	72	*s*_k_^+^	0.95	
			*ω*_k_^+^	0.91	
**e**	*Aspergillus niger*	72	*s*_k_^+^	0.88	
**f**	*Aspergillus niger*	48	*s*_k_^−^	0.93	
		72	*s*_k_^−^	0.85	
	*Aspergillus fumigatus*	48	*s*_k_^−^	0.75	
**g**	*Aspergillus niger*	48	*s*_k_^−^	0.90	
		72	*s*_k_^−^	0.83	
	*Aspergillus fumigatus*	48	*s*_k_^−^	0.74	
**h**	*Aspergillus fumigatus*	48	*s*_k_^0^	0.82	
			*ω*_k_^0^	0.72	
**i**	*Aspergillus fumigatus*	48	*s*_k_^0^	0.80	
**j**	*Candida albicans*	48	*ω*_k_^+^	0.75	
	*Candida glabrata*	24		0.75	
**k**	*Candida albicans*	48	*ω*_k_^+^	0.75	
	*Candida glabrata*	24		0.75	
**l**	*Candida albicans*	48	*ω*_k_^+^	0.75	
	*Candida glabrata*	24		0.75	
**m**	*Candida albicans*	48	*ω*_k_^−^	0.92	
	*Candida glabrata*	24	*ω*_k_^−^	0.92	
	*Candida parapsilosis*	24	*s*_k_^−^	0.78	
**n**	*Candida albicans*	48	*ω*_k_^−^	0.92	
	*Candida glabrata*	24	*ω*_k_^−^	0.92	
	*Candida parapsilosis*	24	*s*_k_^−^	0.77	
**o**	*Candida albicans*	48	*s*_k_^−^	0.83	
	*Candida glabrata*	24		0.83	
	*Candida tropicalis*	48		0.77	
**p**	*Candida parapsilosis*	24	*s*_k_^−^	0.76	
			*ω*_k_^−^	0.76	
**q**	*Candida albicans*	48	*ω*_k_^−^	0.86	
	*Candida glabrata*	24		0.86	
**r**	*Candida albicans*	48	*s*_k_^−^	0.80	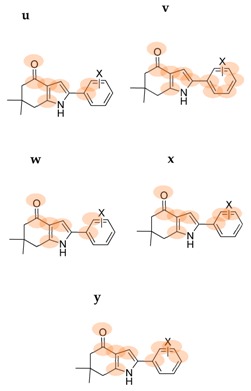
	*Candida glabrata*	24		0.80	
**s**	*Candida albicans*	48	*s*_k_^−^	0.76	
	*Candida glabrata*	24		0.76	
**t**	*Candida albicans*	48	*s*_k_^−^	0.79	
	*Candida glabrata*	24		0.79	
**u**	*Candida albicans*	48	*ω*_k_^−^	0.86	
	*Candida glabrata*	24		0.86	
**v**	*Candida albicans*	48	*s*_k_^−^	0.79	
	*Candida glabrata*	24		0.79	
**w**	*Candida albicans*	48	*ω*_k_^−^	0.86	
	*Candida glabrata*	24		0.86	
**x**	*Candida albicans*	48	*ω*_k_^−^	0.83	
	*Candida glabrata*	24		0.83	
**y**	*Candida albicans*	48	*s*_k_^−^	0.76	
	*Candida glabrata*	24		0.76	
**z**	*Candida albicans*	24	*ω*_k_^0^	0.78	
	*Candida krusei*	24	*ω*_k_^0^	0.78	
		*48*	*ω*_k_^0^	0.78	
	*Candida tropicalis*	24	*ω*_k_^0^	0.78	
		*48*	*ω*_k_^0^	0.76	
	*Candida guilliermondii*	24	*ω*_k_^0^	0.78	
	*Candidad parapsilosis*	48	*s*_k_^0^	0.74	

**Table 7:**
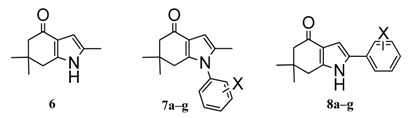
Dipole Moment of indol-4-one; series I compounds **6** and **7a**–**g** and series II compounds **8a**–**g**.

Compound	*µ* (D)
**6**	5.98
**7a**	6.70
**7b**	7.26
**7c**	4.86
**7d**	5.49
**7e**	7.25
**7f**	4.73
**7g**	5.40
**8a**	5.49
**8b**	6.69
**8c**	5.30
**8d**	4.00
**8e**	4.47
**8f**	5.31
**8g**	4.41

## Supplementary Materials

Supplementary materials are available online.
