# Supplementary material for: Theoretical Reactivity Study of Indol-4-Ones and Their Correlation with Antifungal Activity
Source: Molecules. 2017 Mar 8;22(3):427. doi: 10.3390/molecules22030427 (PMC6155404; doi:10.3390/molecules22030427)
Supplement: Supplementary file 1 [file molecules-22-00427-s001.pdf]

Article

Supplementary materials:

María de los Ángeles Zermeño-Macías, Marco Martín González-Chávez, Francisco Méndez, Rodolfo González-Chávez and Arlette Richaud

The cartesian coordinates of compound 6

C	2.00580000	-0.50730000	0.05160000
C	0.82020000	-1.37230000	-0.46070000
C	-0.47620000	-0.66160000	-0.25470000
C	-0.64780000	0.70600000	-0.11960000
C	0.49080000	1.61410000	-0.15250000
C	1.83580000	0.94580000	-0.46080000
H	0.96490000	-1.57900000	-1.53040000
H	0.82340000	-2.34450000	0.04660000
H	1.94030000	0.95680000	-1.55470000
H	2.62430000	1.59030000	-0.06420000
C	2.04740000	-0.52190000	1.59230000
H	2.20440000	-1.53880000	1.96570000
H	2.87070000	0.09800000	1.95830000
H	1.12270000	-0.13990000	2.03090000
C	3.32260000	-1.08770000	-0.48970000
H	4.17890000	-0.50580000	-0.13680000
H	3.46290000	-2.12100000	-0.15560000
H	3.34060000	-1.08100000	-1.58380000
O	0.40860000	2.82140000	0.01320000
N	-1.71240000	-1.23500000	-0.16990000
C	-2.04950000	0.95310000	0.05740000
C	-2.69010000	-0.25740000	0.02060000
C	-4.13370000	-0.61750000	0.14770000
H	-1.89510000	-2.22500000	-0.22340000
H	-2.51340000	1.91710000	0.19420000
H	-4.72640000	0.28540000	0.29910000
H	-4.51320000	-1.11690000	-0.75080000
H	-4.31740000	-1.28170000	0.99980000

The cartesian coordinates of compound 7a

C	2.30430000	-1.51980000	0.13870000
C	0.84570000	-1.24070000	-0.32040000
C	0.52180000	0.21230000	-0.19960000
C	1.43990000	1.24810000	-0.16900000
C	2.87170000	0.99530000	-0.25850000
C	3.25160000	-0.47000000	-0.49430000
H	0.73390000	-1.56290000	-1.36510000
H	0.14630000	-1.84980000	0.26220000
H	3.27180000	-0.61230000	-1.58380000
H	4.27660000	-0.60830000	-0.14070000
C	2.39970000	-1.45580000	1.67610000
H	1.76000000	-2.21590000	2.13550000
H	3.42680000	-1.64150000	2.00340000
H	2.09860000	-0.48050000	2.06560000
C	2.71530000	-2.92580000	-0.32810000
H	3.73530000	-3.15930000	-0.00920000
H	2.05200000	-3.68830000	0.09300000
H	2.67700000	-3.01040000	-1.41840000

O	3.72500000	1.86700000	-0.18880000
N	-0.73290000	0.76020000	-0.07400000
C	0.71200000	2.47060000	-0.01840000
C	-0.61970000	2.15890000	0.03700000
C	-1.81600000	3.04660000	0.15020000
H	1.13520000	3.46160000	0.03140000
H	-1.48950000	4.08730000	0.13470000
H	-2.51570000	2.90040000	-0.67920000
H	-2.37440000	2.88530000	1.07770000
C	-1.95990000	0.02330000	-0.05960000
C	-2.44300000	-0.54750000	-1.23800000
C	-2.66970000	-0.12730000	1.13270000
C	-3.63350000	-1.27160000	-1.22110000
H	-1.88840000	-0.41510000	-2.15950000
C	-3.86410000	-0.84440000	1.14250000
H	-2.27890000	0.30970000	2.04410000
C	-4.34660000	-1.41910000	-0.03250000
H	-4.00580000	-1.71450000	-2.13780000
H	-4.41310000	-0.96030000	2.07000000
H	-5.27400000	-1.98010000	-0.02150000

The cartesian coordinates of compound 7b

C	2.44430000	-1.50780000	0.17660000
C	1.00520000	-1.26530000	-0.35910000
C	0.65960000	0.18660000	-0.30160000
C	1.56110000	1.23460000	-0.26770000
C	2.99910000	0.99810000	-0.28900000
C	3.40650000	-0.46930000	-0.45270000
H	0.94800000	-1.62450000	-1.39610000
H	0.28750000	-1.86400000	0.21220000
H	3.47750000	-0.65130000	-1.53410000
H	4.41610000	-0.58070000	-0.04920000
C	2.46720000	-1.38590000	1.71310000
H	1.81910000	-2.13990000	2.17050000
H	3.48090000	-1.54230000	2.09290000
H	2.13160000	-0.40320000	2.05160000
C	2.89340000	-2.92430000	-0.21830000
H	3.90080000	-3.13070000	0.15440000
H	2.22180000	-3.68000000	0.20220000
H	2.90530000	-3.05090000	-1.30530000
O	3.83690000	1.88420000	-0.21780000
N	-0.60690000	0.72430000	-0.24620000
C	0.81370000	2.45270000	-0.18450000
C	-0.51460000	2.12780000	-0.17120000
C	-1.72720000	2.99630000	-0.10630000
H	1.22400000	3.44930000	-0.14290000
H	-1.41930000	4.04220000	-0.13310000
H	-2.40380000	2.82360000	-0.94980000
H	-2.29710000	2.83870000	0.81480000
C	-1.81760000	-0.02340000	-0.27940000
C	-2.24720000	-0.64890000	-1.45220000
C	-2.60580000	-0.16090000	0.86410000
C	-3.42580000	-1.38980000	-1.47310000
H	-1.64510000	-0.53850000	-2.34630000
C	-3.78680000	-0.88640000	0.86240000
C	-4.19550000	-1.50550000	-0.31640000
H	-3.74500000	-1.86840000	-2.39090000
H	-4.36120000	-0.96190000	1.77730000
H	-5.11570000	-2.07740000	-0.32800000
F	-2.20550000	0.42730000	2.01090000

The cartesian coordnates of compound 7c

C	2.43190000	-1.69760000	0.12260000
C	1.01380000	-1.24910000	-0.32850000
C	0.85950000	0.23090000	-0.19470000
C	1.89060000	1.15350000	-0.16230000
C	3.28380000	0.73840000	-0.26050000
C	3.49170000	-0.75910000	-0.50710000
H	0.86380000	-1.54850000	-1.37530000
H	0.25180000	-1.77970000	0.25250000
H	3.49170000	-0.89560000	-1.59750000
H	4.49510000	-1.01690000	-0.15880000
C	2.53820000	-1.65790000	1.66020000
H	1.82000000	-2.34740000	2.11500000
H	3.53930000	-1.95810000	1.98250000
H	2.34650000	-0.65890000	2.05860000
C	2.67750000	-3.13790000	-0.35660000
H	3.66450000	-3.49010000	-0.04280000
H	1.93200000	-3.82290000	0.06040000
H	2.62780000	-3.20920000	-1.44750000
O	4.23100000	1.50650000	-0.18960000
N	-0.32320000	0.92020000	-0.06180000
C	1.30970000	2.45140000	-0.00310000
C	-0.04890000	2.29590000	0.05510000
C	-1.13510000	3.31310000	0.18580000
H	1.84600000	3.38560000	0.05250000
H	-0.69160000	4.30960000	0.18580000
H	-1.84870000	3.26200000	-0.64310000
H	-1.70600000	3.20180000	1.11320000
C	-1.62710000	0.33220000	-0.05090000
C	-2.20640000	-0.10180000	-1.24440000
C	-2.32110000	0.18990000	1.15210000
C	-3.47210000	-0.68250000	-1.24130000
H	-1.66530000	0.02060000	-2.17490000
C	-3.59080000	-0.38160000	1.16740000
C	-4.13930000	-0.80830000	-0.03250000
H	-3.94100000	-1.02700000	-2.15430000
H	-4.14750000	-0.50290000	2.08800000
F	-5.37090000	-1.36810000	-0.02310000
H	-1.86150000	0.52130000	2.07550000

The cartesian coordinates of compound 7d

C	2.53830000	-1.70030000	0.12470000
C	1.12930000	-1.27920000	-0.37910000
C	0.96120000	0.20240000	-0.29580000
C	1.98080000	1.13530000	-0.26440000
C	3.38020000	0.72990000	-0.31490000
C	3.60680000	-0.77280000	-0.50660000
H	1.00820000	-1.61320000	-1.41930000
H	0.35710000	-1.79720000	0.20000000
H	3.63770000	-0.94410000	-1.59180000
H	4.60250000	-1.01000000	-0.12340000
C	2.60130000	-1.61000000	1.66250000
H	1.87830000	-2.29270000	2.11950000
H	3.59640000	-1.88840000	2.02110000
H	2.38720000	-0.60190000	2.02450000
C	2.80810000	-3.15260000	-0.30170000
H	3.78910000	-3.48590000	0.04920000
H	2.05750000	-3.83080000	0.11720000
H	2.78820000	-3.25840000	-1.39060000

O	4.31780000	1.50940000	-0.24690000
N	-0.23150000	0.88670000	-0.21820000
C	1.38520000	2.43270000	-0.15750000
C	0.02790000	2.26830000	-0.12900000
C	-1.07260000	3.27190000	-0.02720000
H	1.91290000	3.37210000	-0.10890000
H	-0.64360000	4.27460000	-0.03740000
H	-1.77750000	3.20020000	-0.86210000
H	-1.64530000	3.16420000	0.89990000
C	-1.52330000	0.29100000	-0.23720000
C	-2.09980000	-0.15080000	-1.42990000
C	-2.25240000	0.12590000	0.94190000
C	-3.35880000	-0.74320000	-1.44880000
H	-1.54530000	-0.02000000	-2.35120000
C	-3.51200000	-0.45290000	0.96210000
C	-4.03850000	-0.87890000	-0.24750000
H	-3.81180000	-1.08840000	-2.36880000
H	-4.05710000	-0.57000000	1.88910000
F	-5.26250000	-1.44700000	-0.24810000
F	-1.71820000	0.53660000	2.10640000

The cartesian coordinates of compound 7e

C	2.51030000	-1.52460000	0.04190000
C	1.08650000	-1.23950000	-0.51320000
C	0.75530000	0.21140000	-0.39530000
C	1.66380000	1.24860000	-0.28690000
C	3.09940000	0.99760000	-0.27770000
C	3.49700000	-0.46410000	-0.50790000
H	1.04610000	-1.54700000	-1.56770000
H	0.35000000	-1.85560000	0.01410000
H	3.59310000	-0.58990000	-1.59550000
H	4.49530000	-0.60710000	-0.08630000
C	2.49800000	-1.48410000	1.58280000
H	1.82720000	-2.24940000	1.98520000
H	3.49990000	-1.67680000	1.97730000
H	2.16960000	-0.51490000	1.96450000
C	2.95560000	-2.92260000	-0.41750000
H	3.95190000	-3.15900000	-0.03310000
H	2.26720000	-3.69230000	-0.05370000
H	2.99190000	-2.99090000	-1.50920000
O	3.94370000	1.86910000	-0.13800000
N	-0.50680000	0.75820000	-0.36500000
C	0.92320000	2.46920000	-0.17960000
C	-0.40770000	2.15630000	-0.23000000
C	-1.61730000	3.02940000	-0.17010000
H	1.33920000	3.45930000	-0.07850000
H	-1.30350000	4.06950000	-0.07320000
H	-2.23210000	2.94680000	-1.07270000
H	-2.25500000	2.79020000	0.68690000
C	-1.72300000	0.02390000	-0.47990000
C	-2.20550000	-0.32240000	-1.74440000
C	-2.45250000	-0.36960000	0.64900000
C	-3.38700000	-1.04320000	-1.88460000
H	-1.63680000	-0.01310000	-2.61370000
C	-3.63870000	-1.08740000	0.51700000
C	-4.10320000	-1.42330000	-0.75140000
H	-3.74710000	-1.30410000	-2.87270000
H	-4.18530000	-1.37970000	1.40450000
H	-5.02580000	-1.98360000	-0.84970000
Cl	-1.87490000	0.02960000	2.25410000

The cartesian coordinates of compound 7f

C	2.62170000	-1.81380000	0.14550000
C	1.24600000	-1.26150000	-0.32220000
C	1.20690000	0.22750000	-0.20670000
C	2.30690000	1.06640000	-0.17660000
C	3.66420000	0.54180000	-0.26080000
C	3.75520000	-0.96980000	-0.48970000
H	1.08230000	-1.56160000	-1.36650000
H	0.43990000	-1.72330000	0.25800000
H	3.74970000	-1.11900000	-1.57860000
H	4.73350000	-1.30150000	-0.13290000
C	2.72190000	-1.75980000	1.68290000
H	1.94720000	-2.38120000	2.14290000
H	3.69320000	-2.13590000	2.01650000
H	2.61020000	-0.74230000	2.06440000
C	2.75630000	-3.27540000	-0.31210000
H	3.71180000	-3.69790000	0.01220000
H	1.95820000	-3.89420000	0.11080000
H	2.70600000	-3.35900000	-1.40210000
O	4.66780000	1.23430000	-0.18990000
N	0.08120000	1.00870000	-0.08000000
C	1.82980000	2.40690000	-0.02700000
C	0.46320000	2.35980000	0.03020000
C	-0.53760000	3.46330000	0.14280000
H	2.43760000	3.29670000	0.02120000
H	-0.01540000	4.42050000	0.11660000
H	-1.25820000	3.45000000	-0.68140000
H	-1.10970000	3.42160000	1.07520000
C	-1.26310000	0.52430000	-0.06180000
C	-1.84540000	0.03170000	-1.23040000
C	-1.99780000	0.53880000	1.12460000
C	-3.15160000	-0.44990000	-1.21650000
H	-1.27680000	0.03250000	-2.15260000
C	-3.30780000	0.06850000	1.14660000
C	-3.87230000	-0.42450000	-0.02630000
H	-3.60750000	-0.83300000	-2.12040000
H	-3.88090000	0.07640000	2.06470000
Cl	-5.52180000	-1.02590000	-0.00330000
H	-1.53900000	0.91020000	2.03310000

The cartesian coordinates of compound 7g

C	2.75590000	-1.84440000	0.11050000
C	1.37790000	-1.34030000	-0.40290000
C	1.32770000	0.15220000	-0.40310000
C	2.41700000	1.00320000	-0.42760000
C	3.78030000	0.48680000	-0.45790000
C	3.88680000	-1.03660000	-0.57440000
H	1.21680000	-1.71990000	-1.42170000
H	0.57510000	-1.76270000	0.21120000
H	3.89260000	-1.26340000	-1.64980000
H	4.86460000	-1.33230000	-0.18620000
C	2.84530000	-1.68130000	1.64100000
H	2.07280000	-2.27500000	2.13950000
H	3.81770000	-2.02480000	2.00540000
H	2.72230000	-0.64120000	1.95110000
C	2.90690000	-3.33300000	-0.24270000
H	3.86340000	-3.72330000	0.11660000
H	2.11090000	-3.92780000	0.21700000
H	2.86540000	-3.49200000	-1.32460000

O	4.77640000	1.19270000	-0.43190000
N	0.19250000	0.93040000	-0.37090000
C	1.92390000	2.34700000	-0.40500000
C	0.55780000	2.29080000	-0.37200000
C	-0.46210000	3.38060000	-0.34330000
H	2.52250000	3.24420000	-0.41510000
H	0.04490000	4.34540000	-0.38450000
H	-1.15000000	3.32720000	-1.19370000
H	-1.06480000	3.35850000	0.57060000
C	-1.14200000	0.43470000	-0.36130000
C	-1.75630000	0.06760000	-1.56030000
C	-1.86610000	0.29480000	0.82960000
C	-3.05460000	-0.42850000	-1.58680000
H	-1.19800000	0.18020000	-2.48200000
C	-3.16920000	-0.19610000	0.82510000
C	-3.74850000	-0.55400000	-0.38700000
H	-3.52170000	-0.71020000	-2.52110000
H	-3.71600000	-0.29880000	1.75210000
Cl	-5.38740000	-1.17520000	-0.39580000
Cl	-1.13970000	0.72810000	2.35910000

The cartesian coordinates of compound 8a

C	3.42100000	-0.68400000	0.01690000
C	2.16060000	-1.32520000	-0.62960000
C	0.95220000	-0.49160000	-0.36100000
C	0.94380000	0.86580000	-0.07450000
C	2.18630000	1.62230000	0.03120000
C	3.45100000	0.82990000	-0.31650000
H	2.31890000	-1.40970000	-1.71370000
H	2.02760000	-2.34650000	-0.25390000
H	3.60420000	0.95730000	-1.39730000
H	4.29420000	1.31830000	0.17830000
C	3.39540000	-0.88750000	1.54460000
H	3.40980000	-1.95310000	1.79460000
H	4.27250000	-0.42560000	2.00660000
H	2.50720000	-0.44620000	2.00270000
C	4.67780000	-1.35710000	-0.55790000
H	5.58340000	-0.93490000	-0.11290000
H	4.67660000	-2.43240000	-0.35180000
H	4.74220000	-1.22120000	-1.64170000
O	2.24150000	2.80360000	0.33340000
N	-0.34180000	-0.91520000	-0.36060000
C	-0.41620000	1.26630000	0.09320000
C	-1.20420000	0.15190000	-0.09430000
H	-0.75570000	2.25530000	0.35540000
H	-0.64490000	-1.84550000	-0.60210000
C	-2.65530000	-0.02420000	-0.02870000
C	-3.51150000	1.06190000	-0.27720000
C	-3.23450000	-1.26430000	0.28770000
C	-4.89170000	0.91270000	-0.20490000
H	-3.08710000	2.02270000	-0.54360000
C	-4.61760000	-1.41440000	0.34610000
H	-2.60430000	-2.11540000	0.52370000
C	-5.45380000	-0.32700000	0.10230000
H	-5.53250000	1.76500000	-0.40100000
H	-5.04140000	-2.38060000	0.59620000
H	-6.53030000	-0.44270000	0.15170000

The cartesian coordinates of compound 8b

C	3.62960000	-0.66990000	0.01360000
C	2.38770000	-1.35330000	-0.62510000
C	1.15320000	-0.56260000	-0.34640000
C	1.09940000	0.79280000	-0.05570000
C	2.31690000	1.59230000	0.04350000
C	3.60500000	0.84460000	-0.31780000
H	2.54050000	-1.42950000	-1.71050000
H	2.29260000	-2.37940000	-0.25090000
H	3.74320000	0.97850000	-1.39970000
H	4.43520000	1.36110000	0.17020000
C	3.62210000	-0.87640000	1.54130000
H	3.67690000	-1.94120000	1.78890000
H	4.48510000	-0.38360000	1.99760000
H	2.72200000	-0.46830000	2.00690000
C	4.90480000	-1.29840000	-0.57110000
H	5.79800000	-0.84490000	-0.13220000
H	4.94270000	-2.37320000	-0.36570000
H	4.95650000	-1.15950000	-1.65520000
O	2.33430000	2.77240000	0.35210000
N	-0.12340000	-1.03110000	-0.34310000
C	-0.27120000	1.14850000	0.11500000
C	-1.02300000	0.00630000	-0.07000000
H	-0.64690000	2.12730000	0.35710000
H	-0.38920000	-1.97150000	-0.58850000
C	-2.45850000	-0.24990000	0.01110000
C	-3.40890000	0.77450000	-0.10380000
C	-2.97770000	-1.54440000	0.20590000
C	-4.77390000	0.55660000	-0.04620000
C	-4.34500000	-1.79050000	0.26330000
H	-2.29540000	-2.37540000	0.34460000
C	-5.25090000	-0.73920000	0.13680000
H	-5.44180000	1.40290000	-0.14860000
H	-4.70120000	-2.80180000	0.41920000
H	-6.31780000	-0.92050000	0.18510000
F	-2.98550000	2.04800000	-0.28890000

The cartesian coordinates of compound 8c

C	3.80970000	-0.71710000	0.01760000
C	2.54240000	-1.33170000	-0.64130000
C	1.34580000	-0.48240000	-0.36920000
C	1.35700000	0.87120000	-0.06760000
C	2.61080000	1.60730000	0.05310000
C	3.86450000	0.79980000	-0.29870000
H	2.70430000	-1.40700000	-1.72560000
H	2.39190000	-2.35470000	-0.27710000
H	4.02370000	0.93680000	-1.37740000
H	4.71320000	1.26960000	0.20470000
C	3.77460000	-0.93710000	1.54290000
H	3.77050000	-2.00540000	1.78100000
H	4.65750000	-0.49500000	2.01310000
H	2.89220000	-0.48600000	2.00270000
C	5.05840000	-1.40300000	-0.55970000
H	5.96850000	-0.99940000	-0.10700000
H	5.04010000	-2.48020000	-0.36510000
H	5.12900000	-1.25660000	-1.64180000
O	2.68150000	2.78380000	0.36970000
N	0.04470000	-0.88600000	-0.37970000
C	0.00250000	1.29140000	0.09930000

C	-0.80130000	0.19180000	-0.10500000
H	-0.32240000	2.28210000	0.37340000
H	-0.27100000	-1.80720000	-0.63970000
C	-2.25560000	0.03890000	-0.04910000
C	-3.09360000	1.13200000	-0.32780000
C	-2.85720000	-1.18420000	0.28950000
C	-4.47710000	1.01460000	-0.26530000
C	-4.24190000	-1.32050000	0.34130000
H	-2.24410000	-2.03980000	0.55030000
C	-5.02810000	-0.21470000	0.06410000
H	-5.12560000	1.85450000	-0.48130000
H	-4.70860000	-2.26080000	0.60710000
H	-2.65440000	2.08070000	-0.61140000
F	-6.37720000	-0.33760000	0.11650000

The cartesian coordinates of compound 8d

C	4.41020000	-0.54880000	-0.16550000
C	3.14660000	-1.01760000	-0.94120000
C	1.94700000	-0.24100000	-0.51050000
C	1.95830000	1.01880000	0.07300000
C	3.21070000	1.71500000	0.34940000
C	4.46480000	1.00080000	-0.16380000
H	3.31250000	-0.87110000	-2.01730000
H	2.99480000	-2.09290000	-0.79310000
H	4.62190000	1.35770000	-1.19130000
H	5.31360000	1.35750000	0.42480000
C	4.37030000	-1.07700000	1.28210000
H	4.36800000	-2.17140000	1.29470000
H	5.25020000	-0.73900000	1.83680000
H	3.48510000	-0.73230000	1.82180000
C	5.66190000	-1.10220000	-0.86580000
H	6.56980000	-0.79770000	-0.33730000
H	5.64450000	-2.19660000	-0.89190000
H	5.73610000	-0.74090000	-1.89600000
O	3.27780000	2.79630000	0.91080000
N	0.65070000	-0.63340000	-0.61320000
C	0.60670000	1.38840000	0.32740000
C	-0.19710000	0.35280000	-0.10490000
H	0.28040000	2.30250000	0.79640000
H	0.33950000	-1.52560000	-0.96350000
C	-1.66050000	0.27460000	-0.11530000
C	-2.38650000	1.45050000	-0.39480000
C	-2.43140000	-0.86770000	0.16560000
C	-3.77140000	1.50050000	-0.38130000
C	-3.82340000	-0.84930000	0.17120000
C	-4.48550000	0.33960000	-0.10040000
H	-4.29160000	2.42320000	-0.60280000
H	-4.37620000	-1.75080000	0.39590000
H	-1.83200000	2.34560000	-0.64660000
F	-1.86570000	-2.04160000	0.47690000
F	-5.82610000	0.36150000	-0.09480000

The cartesian coordinates of compound 8e

C	3.69200000	-0.64760000	-0.10070000
C	2.40840000	-1.12360000	-0.83760000
C	1.24180000	-0.27010000	-0.46590000
C	1.30410000	1.02900000	0.02070000
C	2.58320000	1.69400000	0.24290000

C	3.80690000	0.89420000	-0.21530000
H	2.57620000	-1.06570000	-1.92190000
H	2.21520000	-2.17800000	-0.60850000
H	3.97500000	1.16630000	-1.26680000
H	4.67090000	1.26040000	0.34480000
C	3.63740000	-1.06440000	1.38250000
H	3.59320000	-2.15400000	1.47650000
H	4.53170000	-0.72010000	1.90960000
H	2.76790000	-0.64720000	1.89580000
C	4.91880000	-1.30010000	-0.75870000
H	5.84000000	-0.99210000	-0.25590000
H	4.85920000	-2.39200000	-0.70270000
H	5.00230000	-1.02020000	-1.81320000
O	2.69630000	2.81150000	0.72070000
N	-0.06900000	-0.61740000	-0.53930000
C	-0.03190000	1.46900000	0.24640000
C	-0.87670000	0.43620000	-0.10650000
H	-0.32170000	2.42820000	0.64380000
H	-0.41710000	-1.52190000	-0.81580000
C	-2.34320000	0.41260000	-0.11220000
C	-3.02060000	1.62910000	-0.33760000
C	-3.15670000	-0.71350000	0.10800000
C	-4.40430000	1.72110000	-0.32680000
C	-4.54660000	-0.63840000	0.10700000
C	-5.17680000	0.58160000	-0.10780000
H	-4.87940000	2.67860000	-0.50500000
H	-5.12420000	-1.53640000	0.28470000
H	-2.42700000	2.51130000	-0.54200000
H	-6.25880000	0.63620000	-0.10650000
Cl	-2.46510000	-2.31050000	0.44490000

The cartesian coordinates of compound 8f

C	4.20450000	-0.74460000	0.03130000
C	2.93250000	-1.34800000	-0.62910000
C	1.74520000	-0.48280000	-0.36700000
C	1.77190000	0.87290000	-0.07450000
C	3.03440000	1.59460000	0.04630000
C	4.27940000	0.76940000	-0.29490000
H	3.09740000	-1.43140000	-1.71230000
H	2.76840000	-2.36700000	-0.25960000
H	4.44470000	0.89740000	-1.37380000
H	5.13180000	1.23190000	0.20900000
C	4.16030000	-0.95390000	1.55770000
H	4.13980000	-2.02040000	1.80310000
H	5.04770000	-0.52140000	2.02840000
H	3.28280000	-0.48690000	2.01090000
C	5.44700000	-1.44970000	-0.53640000
H	6.36010000	-1.05430000	-0.08250000
H	5.41460000	-2.52530000	-0.33480000
H	5.52380000	-1.31140000	-1.61920000
O	3.11770000	2.77220000	0.35520000
N	0.43990000	-0.87070000	-0.38050000
C	0.42260000	1.31070000	0.08280000
C	-0.39430000	0.21920000	-0.11750000
H	0.10910000	2.30760000	0.34760000
H	0.11500000	-1.79050000	-0.63450000
C	-1.84910000	0.08490000	-0.06490000
C	-2.67410000	1.19420000	-0.31530000
C	-2.46940000	-1.13710000	0.24110000
C	-4.05830000	1.09230000	-0.25810000
C	-3.85520000	-1.25510000	0.28840000

C	-4.64210000	-0.13650000	0.03900000
H	-4.68130000	1.95540000	-0.45540000
H	-4.31870000	-2.20290000	0.53060000
H	-2.22520000	2.14570000	-0.57380000
H	-1.87130000	-2.00970000	0.48040000
Cl	-6.39360000	-0.27540000	0.09910000

The cartesian coordinates of compound 8g

C	4.41180000	-0.55220000	-0.11290000
C	3.14420000	-1.07380000	-0.84730000
C	1.94730000	-0.26660000	-0.46820000
C	1.96260000	1.03180000	0.02310000
C	3.21690000	1.74440000	0.24320000
C	4.46800000	0.99350000	-0.22290000
H	3.30540000	-1.00530000	-1.93180000
H	2.99190000	-2.13550000	-0.62160000
H	4.62080000	1.27530000	-1.27400000
H	5.31980000	1.39040000	0.33490000
C	4.37780000	-0.97490000	1.36920000
H	4.37440000	-2.06550000	1.46040000
H	5.26050000	-0.59900000	1.89420000
H	3.49540000	-0.59100000	1.88660000
C	5.65970000	-1.15620000	-0.77700000
H	6.57030000	-0.81570000	-0.27590000
H	5.64100000	-2.24960000	-0.72440000
H	5.72960000	-0.87000000	-1.83080000
O	3.28770000	2.86320000	0.72520000
N	0.65020000	-0.66360000	-0.53670000
C	0.61250000	1.42060000	0.25620000
C	-0.19430000	0.35780000	-0.09700000
H	0.28930000	2.36660000	0.65980000
H	0.33640000	-1.57820000	-0.82060000
C	-1.65780000	0.28100000	-0.09530000
C	-2.38180000	1.47110000	-0.31340000
C	-2.43070000	-0.87250000	0.12840000
C	-3.76660000	1.52300000	-0.29520000
C	-3.82250000	-0.85170000	0.13720000
C	-4.48260000	0.35080000	-0.07260000
H	-4.28520000	2.45680000	-0.46870000
H	-4.37690000	-1.76240000	0.31650000
H	-1.82580000	2.37670000	-0.52030000
Cl	-6.23620000	0.38290000	-0.06240000
Cl	-1.68660000	-2.44240000	0.45880000

Parr function

Parr function for nucleophilic P^{+}(r), and electrophilic P(r) attacks were calculated using: $P^{+}(r) = \rho_{s}^{ra}(r)$, and $P^{-}(r) = \rho_{s}^{rc}(r)$; where $\rho_{s}^{ra}(r)$ is the atomic spin density of the radical anion, and $\rho_{s}^{rc}(r)$ is the atomic spin density of the radical cation [41-42].

Table S1. Nucleophilic ***P+* (r)** and electrophilic ***P-*(r)** Parr Function calculated using Mülliken atomic spin densities for series I (**6,** and **7a-g**) and series II (**8a-g**) by fragments.

ID	Microorganism	Time of testing	***P+* (r)** [N+1 Mülliken]			***P-*(r)** [N-1 Mülliken]		
			**r**	**r²**	**p**	**r**	**r²**	**p**
**a**	*A. fumigatus*	48	0.78	0.61	0.0221627	0.80	0.64	0.017802
	A. fumigatus	72	--	--	--	0.87	0.76	0.004754
	A. niger	48	--	--	--	0.93	0.86	0.002741
	A. niger	72	--	--	--	0.82	0.68	0.043257
**b**	*A. niger*	48	0.83	0.69	0.0206901	--	--	--
	A. fumigatus	48	0.77	0.59	0.0258607	--	--	--
	A. fumigatus	72	0.78	0.61	0.0225558	0.82	0.67	0.012901
**c**	*A. niger*	72	0.94	0.89	0.01153733	--	--	--
**d**	*A. fumigatus*	72	---	--	--	0.74	0.54	0.058456
**e**	*A. niger*	48	0.84	0.71	0.0343775	--	--	--
	A. niger	72	0.92	0.84	0.0279788	--	--	--
**k**	*C. parapsilosis*	24	--	--	--	0.78	0.60	0.040351
**l**	*C. albicans*	24	0.81	0.65	0.0289368	--	--	--
	C. krusei	24	0.81	0.65	0.0289368	--	--	--
	C. krusei	48	0.81	0.65	0.0289368	--	--	--
	C. tropicalis	24	0.81	0.65	0.0289368	--	--	--
	C. tropicalis	48	--	--	--	0.77	0.60	0.040861
	C. guilliermondii	24	0.81	0.65	0.0289368	--	--	--
**m**	*C. albicans*	24	0.82	0.68	0.0223158	--	--	--
	C. krusei	24	0.82	0.68	0.0223158	--	--	--
	C. krusei	48	0.82	0.68	0.0223158			
	C. tropicalis	24	0.82	0.68	0.0223158	--	--	--
	C. tropicalis	48	0.79	0.62	0.0358123	--	--	--
	C. guilliermondii	24	0.82	0.68	0.0223158	--	--	--
**n**	*C. parapsilosis*	24	--	--	--	0.87	0.76	0.010948
**o**	*C. albicans*	24	--	--	--	0.79	0.62	0.03622
	C. albicans	48	--	--	--	0.99	0.97	$4.1x10^{-5}$
	C. glabrata	24	--	--	--	0.99	0.97	$4.1x10^{-5}$
	C. krusei	24	--	--	--	0.79	0.62	0.03622
	C. krusei	48	--	--	--	0.79	0.62	0.03622
	C. tropicalis	24	--	--	--	0.79	0.62	0.03622
	C. tropicalis	48	--	--	--	0.88	0.77	0.009136
	C. guilliermondii	24	--	--	--	0.79	0.62	0.03622
	C. guilliermondii	48	--	--	--	0.79	0.62	0.034975
**s**	*C. albicans*	48	--	--	--	0.78	0.62	0.036781
	C. glabrata	24	--	--	--	0.78	0.62	0.036781
	C. tropicalis	48	--	--	--	0.76	0.58	0.046149
**t**	*C. albicans*	24	--	--	--	0.77	0.59	0.043474
	C. albicans	48	--	--	--	0.98	0.96	$9.5x10^{-5}$
	C. glabrata	24	--	--	--	0.98	0.96	$9.5x10^{-5}$
	C. krusei	24	--	--	--	0.77	0.59	0.043474
	C. krusei	48	--	--	--	0.77	0.59	0.043474
	C. tropicalis	24	--	--	--	0.77	0.59	0.043474
	C. tropicalis	48	--	--	--	0.86	0.74	0.012865

	C. guilliermondii	24	--	--	--	0.77	0.59	0.043474
	C. guilliermondii	48	--	--	--	0.80	0.64	0.031035
**u**	*C. albicans*	24	--	--	--	0.80	0.64	0.031157
	C. albicans	48	--	--	--	0.96	0.91	0.000749
	C. glabrata	24	--	--	--	0.96	0.91	0.000749
	C. krusei	24	--	--	--	0.80	0.64	0.031157
	C. krusei	48	--	--	--	0.80	0.64	0.031157
	C. tropicalis	24	--	--	--	0.80	0.64	0.031157
	C. tropicalis	48	--	--	--	0.91	0.82	0.004721
	C. guilliermondii	24	--	--	--	0.80	0.64	0.031157
	C. guilliermondii	48	--	--	--	0.76	0.58	0.047998
**w**	*C. albicans*	24	--	--	--	0.80	0.64	0.031157
	C. albicans	48	--	--	--	0.96	0.91	0.000749
	C. glabrata	24	--	--	--	0.96	0.91	0.000749
	C. krusei	24	--	--	--	0.80	0.64	0.031157
	C. krusei	48	--	--	--	0.80	0.64	0.031157
	C. tropicalis	24	--	--	--	0.80	0.64	0.031157
	C. tropicalis	48	--	--	--	0.91	0.82	0.004721
	C. guilliermondii	24	--	--	--	0.80	0.64	0.031157
	C. guilliermondii	48	--	--	--	0.76	0.58	0.47998
**x**	*C. albicans*	24	--	--	--	0.77	0.60	0.040908
	C. albicans	48	--	--	--	0.98	0.96	$1.43x10^{-4}$
	C. glabrata	24	--	--	--	0.98	0.96	$1.43x10^{-4}$
	C. krusei	24	--	--	--	0.77	0.60	0.040908
	C. krusei	48	--	--	--	0.77	0.60	0.040908
	C. tropicalis	24	--	--	--	0.77	0.60	0.040908
	C. tropicalis	48	--	--	--	0.87	0.75	0.011252
	C. guilliermondii	24	--	--	--	0.77	0.60	0.040908
	C. guilliermondii	48	--	--	--	0.84	0.71	0.0117966
**y**	*C. albicans*	24	--	--	--	0.81	0.65	0.027947
	C. albicans	48	--	--	--	0.98	0.96	$9.4x10^{-5}$
	C. glabrata	24	--	--	--	0.98	0.96	$9.4x10^{-5}$
	C. krusei	24	--	--	--	0.81	0.65	0.027947
	C. krusei	48	--	--	--	0.81	0.65	0.027947
	C. tropicalis	24	--	--	--	0.81	0.65	0.027947
	C. tropicalis	48	--	--	--	0.91	0.82	0.00467
	C. guilliermondii	24	--	--	--	0.81	0.65	0.027947
	C. guilliermondii	48	--	--	--	0.82	0.67	0.0236

Fragments are showed in Table 6 of this paper.
The spaces without numerical values does not have statistical significance.

Table S2. Nucleophilic ***P⁺*** **(r)** and electrophilic ***P⁻***(r) Parr Function calculated using Hirshfeld atomic spin densities for series I (**6,** and **7a-g**) and series II (**8a-g**) by fragments.

ID	Microorganism	Time of testing	P^{+} (r) [N+1 Hirshfeld]			P^{-}(r) [N-1 Hirshfeld]		
			r	r^2	p	r	r^2	p
a	*A. fumigatus*	48	0.82	0.67	0.02379836	0.80	0.64	0.017872
	A. fumigatus	72	--	--	--	0.87	0.76	0.0045075
	A. niger	48	--	--	--	0.93	0.86	0.0025894
	A. niger	72	--	--	--	0.82	0.68	0.0446142
b	*A. fumigatus*	48	0.84	0.70	0.0098806	0.81	0.65	0.0155901
	A. fumigatus	72	--	--	--	0.86	0.75	0.0056491
	A. niger	48	--	--	--	0.92	0.86	0.0027815
	A. niger	72	--	--	--	0.84	0.70	0.0380387
	A. niger	48	--	--	--	0.80	0.64	0.0543112
c	*A. niger*	72	0.99	0.98	0.00161952	--	--	--
	A. niger	48	--	--	--	0.82	0.67	0.0467549
d	*A. niger*	72	0.97	0.94	0.00656398	--	--	--
	A. niger	48	--	--	--	0.82	0.67	0.047405
e	*A. niger*	72	0.92	0.84	0.02722828	--	--	--
j	*C. albicans*	48	0.76	0.57	0.0491557	--	--	--
	C. glabrata	24	0.76	0.57	0.0491557	--	--	--
	C. tropicalis	48	--	--	--	0.75	0.57	0.049812
l	*C. albicans*	24	--	--	--	0.89	0.79	0.007048
	C. krusei	24	--	--	--	0.89	0.79	0.007048
	C. krusei	48	--	--	--	0.89	0.79	0.007048
	C. tropicalis	24	--	--	--	0.89	0.79	0.007048
	C. tropicalis	48	--	--	--	0.90	0.81	0.005792
	C. guilliermondii	24	--	--	--	0.89	0.79	0.007048
	C. parapsilosis	24	--	--	--	0.86	0.74	0.013318
	C. parapsilosis	24	--	--	--	0.87	0.76	0.010548
o	*C. albicans*	24	--	--	--	0.74	0.55	0.057644
	C. albicans	48	--	--	--	0.99	0.98	$3.12x10^{-5}$
	C. glabrata	24	--	--	--	0.99	0.98	$3.12x10^{-5}$
	C. krusei	24	--	--	--	0.74	0.55	0.057644
	C. krusei	48	--	--	--	0.74	0.55	0.057644
	C. tropicalis	24	--	--	--	0.74	0.55	0.057644
	C. tropicalis	48	--	--	--	0.85	0.72	0.015656
	C. guilliermondii	24	--	--	--	0.74	0.55	0.057644
	C. guilliermondii	48	--	--	--	0.86	0.74	0.013513
s	*C. albicans*	48	--	--	--	0.74	0.55	0.056886
t	*C. glabrata*	24	--	--	--	0.74	0.55	0.056886
	C. albicans	48	--	--	--	0.96	0.92	0.000653
	C. glabrata	24	--	--	--	0.96	0.92	0.000653
	C. tropicalis	48	--	--	--	0.79	0.62	0.035976
	C. guilliermondii	48	--	--	--	0.82	0.67	0.024104
u	*C. albicans*	24	--	--	--	0.82	0.67	0.024741
	C. albicans	48	--	--	--	0.85	0.73	0.014287
	C. glabrata	24	--	--	--	0.85	0.73	0.014287
	C. krusei	24	--	--	--	0.82	0.67	0.024744
	C. krusei	48	--	--	--	0.82	0.67	0.024744
	C. tropicalis	24	--	--	--	0.82	0.67	0.024744
	C. tropicalis	48	--	--	--	0.87	0.76	0.01058
	C. guilliermondii	24	--	--	--	0.82	0.67	0.024744
	C. guilliermondii	48	--	--	--	0.75	0.56	0.053734
	C. parapsilosis	48	--	--	--	0.78	0.61	0.039073
w	*C. albicans*	24	--	--	--	0.82	0.67	0.024744

	C. albicans	48	--	--	--	0.85	0.73	0.014287
	C. glabrata	24	--	--	--	0.85	0.73	0.014287
	C. krusei	24	--	--	--	0.82	0.67	0.024744
	C. krusei	48	--	--	--	0.82	0.67	0.024744
	C. tropicalis	24	--	--	--	0.82	0.67	0.024744
	C. tropicalis	48	--	--	--	0.87	0.76	0.01058
	C. guilliermondii	24	--	--	--	0.82	0.67	0.024744
	C. guilliermondii	48	--	--	--	0.75	0.56	0.053734
**x**	*C. albicans*	48	--	--	--	0.76	0.57	0.049039
	C. glabrata	24	--	--	--	0.76	0.57	0.049039
	C. guilliermondii	48	--	--	--	0.76	0.58	0.045066
**y**	*C. albicans*	24	--	--	--	0.83	0.69	0.020166
	C. albicans	48	--	--	--	0.98	0.96	$9.44x10^{-5}$
	C. glabrata	24	--	--	--	0.98	0.96	$9.44x10^{-5}$
	C. krusei	24	--	--	--	0.83	0.69	0.020166
	C. krusei	48	--	--	--	0.83	0.69	0.020166
	C. tropicalis	24	--	--	--	0.83	0.69	0.020166
	C. tropicalis	48	--	--	--	0.91	0.83	0.004042
	C. guilliermondii	24	--	--	--	0.83	0.69	0.020166
	C. guilliermondii	48	--	--	--	0.87	0.76	0.010494

Fragments are showed in Table 6 of this paper.
The spaces without numerical values does not have statistical significance

Table S3. Representative m, b, r, r^2 of equations of each statistical analysis.

**SIMPLE LINEAL REGRESSION FOR SERIES I**							
**Microorganism**	**Time of testing**	**Chemical reactivity parameter**	**m**	**b**	**r**	**r^2**	**p**
C. glabrata	48	***η***	6.72	-22.52	0.98	0.96	0.00002
C. guilliermondii	24		2.89	-10.76	0.96	0.92	0.0002
C. albicans	48	χ	-6.33	25.26	0.77	0.60	0.0246
C. krusei	48		-15.84	62.04	0.75	0.57	0.0302
C. albicans	48	ω	-4.85	10.23	0.83	0.70	0.0099
C. glabrata	48		-5.23	13.25	0.86	0.75	0.005498
**SIMPLE LINEAL REGRESSION FOR SERIES II**							
**Microorganism**	**Time of testing**	**Chemical reactivity parameter**	**m**	**b**	**r**	**r^2**	**p**
C. albicans	48	***η***	1.29	-3.96	0.78	0.60	0.0406
C. glabrata	24		0.65	-1.98	0.78	0.60	0.0406
C. albicans	48	χ	-0.73	3.63	0.98	0.97	0.00006
C. glabrata	24		-0.36	1.82	0.98	0.97	0.00006
C. albicans	48	ω	-0.62	2.06	0.98	0.95	0.0002
C. glabrata	24		-0.31	1.03	0.98	0.95	0.0002
**MULTIPLE REGRESSION**							
**Microorganism**	**Time of testing**	**Chemical reactivity parameter**	**m**	**b**	**r**	**r^2**	**p**
C. guilliermondii	24	***η***<br>S	32.64<br>477.1	-249.2	0.99	0.99	<0.0001
	48	***η***<br>S	90.50<br>1339	-695.8	0.99	0.99	<0.0001
**SIMPLE LINEAL REGRESSION BY FRAGMENT**							
**Microorganism**	**Time of testing**	**Chemical reactivity parameter**	**m**	**b**	**r**	**r^2**	**p**

A. niger	72	Fragmento: c, sf_k^+	-42.66	3.66	0.98	0.96	0.0027
		d, sf_k^+	-34.42	4.27	0.95	0.90	0.0131
C. albicans	48	m, ωf_k^+	-7.29	2.02	0.92	0.86	0.0029
C. glabrata	24		-3.65	1.01	0.92	0.86	0.0029

This table only shows the two equations with higher Pearson coefficient than others equations.

Table S4. Results obtained via multiple regression analysis for series I (**6** and **7a-g**) to *Candida albicans* 24 h.

Dependent variable: ACT

Source	DF	Sum of Squares	Mean Square	F Value	Pr > F
Model	2	1.00070777	0.50035389	42.41	0.0007
Error	5	0.05899575	0.01179915		
Corrected Total	7	1.05970352			

R-Square	Adj R-Sq	Coeff Var	Root MSE	ACT Mean
0.952865	0.9443	22.82871	0.108624	0.475821

Source	DF	TYPE III SS	Mean Square	F Value	Pr > F
D	1	0.21814765	0.21814765	18.49	0.0077
B	1	0.19481674	0.19481674	16.51	0.0097

Parameter	Estimate	Standar Error	t Value	Pr > |t|
Intercept	-419.2394583	100.4227233	-4.17	0.0087
D	53.9017215	12.5358196	4.30	0.0077
B	816.4342169	200.9247462	4.06	0.0097

ACT= antifungal activity, D= hardness, and B= softness.

Table S5. Results obtained via multiple regression analysis for series I (**6** and **7a-g**) to *Candida albicans* 48 h.

Dependent variable: ACT

Source	DF	Sum of Squares	Mean Square	F Value	Pr > F
Model	1	2.73574080	2.73574080	13.77	0.0100
Error	6	1.19188714	0.19864786		
Corrected Total	7	3.92762794			

R-Square	Adj R-Sq	Coeff Var	Root MSE	ACT Mean
0.736149	0.696538	30.07028	0.445699	1.482192

Source	DF	TYPE III SS	Mean Square	F Value	Pr > F
EF	1	2.73574080	2.73574080	13.77	0.0100

Parameter	Estimate	Standar Error	t Value	Pr > |t|
Intercept	10.22897221	2.36222431	4.33	0.0049
EF	-4.84763223	1.30627355	-3.71	0.0100

ACT= antifungal activity and EF= electrophilicity.

Table S6. Results obtained via multiple regression analysis for series I (**6** and **7a-g**) to *Candida glabrata* 24 h

Dependent variable: ACT

Source	DF	Sum of Squares	Mean Square	F Value	Pr > F
Model	2	3.75217205	1.87608602	37.91	0.0010
Error	5	0.24746882	0.04949376		
Corrected Total	7	3.99964086			

R-Square	Adj R-Sq	Coeff Var	Root MSE	ACT Mean
0.951837	0.938127	21.95478	0.222472	1.013319

Source	DF	TYPE III SS	Mean Square	F Value	Pr > F
D	1	0.57492179	0.57492179	11.62	0.0191
B	1	0.49923134	0.49923134	10.09	0.0246

Parameter	Estimate	Standar Error	t Value	Pr > |t|
Intercept	-675.644935	205.6751714	-3.29	0.0218
D	87.504775	25.6745362	3.41	0.0191
B	1306.950033	411.5127559	3.18	0.0246

ACT= antifungal activity, D= hardness, and B= softness.

Table S7. Results obtained via multiple regression analysis for series I (**6** and **7a-g**) to *Candida glabrata* 48 h.

Dependent variable: ACT

Source	DF	Sum of Squares	Mean Square	F Value	Pr > F
Model	1	4.07990010	4.07990010	139.83	<.0001
Error	6	0.17506309	0.02917718		
Corrected Total	7	4.25496319			

R-Square	Adj R-Sq	Coeff Var	Root MSE	ACT Mean
0.978172	0.958857	4.484792	0.170813	3.808723

Source	DF	TYPE III SS	Mean Square	F Value	Pr > F
D	1	4.07990010	4.07990010	139.83	<.0001

Parameter	Estimate	Standar Error	t Value	Pr > |t|
Intercept	-22.51621612	2.22701947	-10.11	<.0001
D	6.71638514	0.56797928	11.83	<.0001

ACT= antifungal activity and D= hardness.

Table S8. Results obtained via multiple regression analysis for series I (**6** and **7a-g**) to *Candida glabrata* 24 h.

Dependent variable: ACT

**Source**	**DF**	**Sum of Squares**	**Mean Square**	**F Value**	**Pr > F**
Model	1	4.18161839	4.18161839	58.42	0.0003
Error	6	0.42945749	0.07157625		
Corrected Total	7	4.61107588			

**R-Square**	**Adj R-Sq**	**Coeff Var**	**Root MSE**	**ACT Mean**
0.944941	0.906864	29.16232	0.267537	0.917408

**Source**	**DF**	**TYPE III SS**	**Mean Square**	**F Value**	**Pr > F**
D	1	4.18161839	4.18161839	58.42	0.0003

**Parameter**	**Estimate**	**Standar Error**	**t Value**	**Pr > |t|**
Intercept	-25.73367184	3.48808302	-7.38	0.0003
D	6.79959469	0.88960106	7.64	0.0003

ACT= antifungal activity and D= hardness.

Table S9. Results obtained via multiple regression analysis for series I (**6** and **7a-g**) to *Candida krusei* 48 h.

Dependent variable: ACT

**Source**	**DF**	**Sum of Squares**	**Mean Square**	**F Value**	**Pr > F**
Model	1	16.56056702	16.56056702	10.83	0.0166
Error	6	9.17397758	1.52899626		
Corrected Total	7	25.73454461			

**R-Square**	**Adj R-Sq**	**Coeff Var**	**Root MSE**	**ACT Mean**
0.663534	0.643515	48.63972	1.236526	2.542214

**Source**	**DF**	**TYPE III SS**	**Mean Square**	**F Value**	**Pr > F**
D	1	16.56056702	16.56056702	10.83	0.0166

**Parameter**	**Estimate**	**Standar Error**	**t Value**	**Pr > |t|**
Intercept	24.06251556	6.55363733	3.67	0.0104
EF	-11.92696079	3.62406019	-3.29	0.0166

ACT= antifungal activity and EF= electrophilicity.

Table S10. Results obtained via multiple regression analysis for series I (**6** and **7a-g**) to *Candida tropicalis 24* h

Dependent variable: ACT

Source	DF	Sum of Squares	Mean Square	F Value	Pr > F
Model	1	1.04540460	1.04540460	58.42	0.0003
Error	6	0.10736437	0.01789406		
Corrected Total	7	1.15276897			

R-Square	Adj R-Sq	Coeff Var	Root MSE	ACT Mean
0.944941	0.906864	29.16232	0.133769	0.458704

Source	DF	TYPE III SS	Mean Square	F Value	Pr > F
D	1	1.04540460	1.04540460	58.42	0.0003

Parameter	Estimate	Standar Error	t Value	Pr > |t|
Intercept	-12.86683592	1.74404151	-7.38	0.0003
D	3.39979735	0.44480053	7.64	0.0003

ACT= antifungal activity and D= hardness.

Table S11. Results obtained via multiple regression analysis for series I (**6** and **7a-g**) to *Candida tropicalis* 48 h.

Dependent variable: ACT

Source	DF	Sum of Squares	Mean Square	F Value	Pr > F
Model	1	19.80422656	19.80422656	9.89	0.0200
Error	6	12.01639911	2.00273319		
Corrected Total	7	31.82062567			

R-Square	Adj R-Sq	Coeff Var	Root MSE	ACT Mean
0.637422	0.622371	60.70790	1.415180	2.331129

Source	DF	TYPE III SS	Mean Square	F Value	Pr > F
EF	1	19.80422656	19.80422656	9.89	0.0200

Parameter	Estimate	Standar Error	t Value	Pr > |t|
Intercept	25.86480113	7.50050906	3.45	0.0137
EF	-13.04280896	4.14766562	-3.14	0.0200

ACT= antifungal activity and EF= electrophilicity.

Table S12. Results obtained via multiple regression analysis for series I (**6** and **7a-g**) to *Candida guilliermondii* 24 h.

Dependent variable: ACT

Source	DF	Sum of Squares	Mean Square	F Value	Pr > F
Model	2	0.82179258	0.41089629	659.21	<.0001
Error	5	0.00311657	0.00062331		
Corrected Total	7	0.82490916			

R-Square	Adj R-Sq	Coeff Var	Root MSE	ACT Mean
0.997518	0.996222	4.424713	0.024966	0.564246

Source	DF	TYPE III SS	Mean Square	F Value	Pr > F
D	1	0.08001138	0.08001138	128.36	<.0001
B	1	0.06652788	106.73	106.73	0.0001

Parameter	Estimate	Standar Error	t Value	Pr > |t|
Intercept	-249.1945678	23.08130725	-10.80	0.0001
D	32.6439914	2.88125132	11.33	<.0001
B	477.1005375	46.18084084	10.33	0.0001

ACT= antifungal activity, D= hardness, and B= softness.

Table S13. Results obtained via multiple regression analysis for series I (**6** and **7a-g**) to *Candida guilliermondii 48* h.

Dependent variable: ACT

Source	DF	Sum of Squares	Mean Square	F Value	Pr > F
Model	2	4.94331842	2.47165921	1280.59	<.0001
Error	5	0.00965045	0.00193009		
Corrected Total	7	4.95296887			

R-Square	Adj R-Sq	Coeff Var	Root MSE	ACT Mean
0.999165	0.998052	5.932391	0.043933	0.740558

Source	DF	TYPE III SS	Mean Square	F Value	Pr > F
D	1	0.61492285	0.61492285	318.60	<.0001
B	1	0.52403184	0.52403184	271.51	<.0001

Parameter	Estimate	Standar Error	t Value	Pr > |t|
Intercept	-695.836387	40.61584049	-17.13	<.0001
D	90.497730	5.07009602	17.85	<.0001
B	1339.019494	81.26375364	16.48	<.0001

ACT= antifungal activity, D= hardness, and B= softness.

Table S14. Results obtained via multiple regression analysis for series I (**6** and **7a-g**) to *Candida parapsilosis* 24 h.

Dependent variable: ACT

Source	DF	Sum of Squares	Mean Square	F Value	Pr > F
Model	4	0.00239754	0.00059939	0.79	0.6023
Error	3	0.00228214	0.00076071		
Corrected Total	7	0.00467968			

R-Square	Adj R-Sq	Coeff Var	Root MSE	ACT Mean
0.512330	--------	43.16949	0.027581	0.063890

Source	DF	TYPE III SS	Mean Square	F Value	Pr > F
D	1	0.00113718	0.00113718	1.49	0.3088
B	1	0.00104182	0.00104182	1.37	0.3264
P	0	0.00000000	…….	…….	…….
EN	0	0.00000000	…….	…….	…….
EF	1	0.00072981	0.00072981	0.96	0.3996

Parameter	Estimate	Standar Error	t Value	Pr > |t|
Intercept	-163.0352483	140.1754891	-1.16	0.3289
D	14.9081494	12.1932610	1.22	0.3088
B	325.1155845	277.8122458	1.17	0.3264
P	-11.6591957 B	11.9917845	-0.97	0.4026
EN	0.0000000 B	……	……	……
EF	-12.2718257	12.5289319	-0.98	0.3996

NOTE: The X'X matrix has been found to be singular, and a generalized inverse was used to solve the normal equations. Terms whose estimates are followed by the letter 'B' are not uniquely estimable.

ACT= antifungal activity, D= hardness, B= softness, P=chemical potential, EN= electronegativity, and EF= electrophilicity.

Table S15. Results obtained via multiple regression analysis for series I (**6** and **7a-g**) to *Candida parapsilosis* 48 h.

Dependent variable: ACT

Source	DF	Sum of Squares	Mean Square	F Value	Pr > F
Model	4	0.15081409	0.03770352	0.24	0.8980
Error	3	0.46743738	0.15581246		
Corrected Total	7	0.61825147			

R-Square	Adj R-Sq	Coeff Var	Root MSE	ACT Mean
0.243936	---------	102.9634	0.394731	0.383370

Source	DF	TYPE III SS	Mean Square	F Value	Pr > F
D	1	0.01704740	0.01704740	0.11	0.7626
B	1	0.02039629	0.02039629	0.13	0.7415
P	0	0.00000000	. . .	. . .	
EN	0	0.00000000	. . .	. . .	
EF	1	0.02641098	0.02641098	0.17	0.7082

Parameter	Estimate	Standar Error	t Value	Pr > |t|
Intercept	719.718357	2006.148330	0.36	0.7435
D	-57.721667	174.506188	-0.33	0.7626
B	-1438.523113	3975.963106	-0.36	0.7415

P	68.945684 B	171.622718	0.40	0.7148
EN	0.000000 B	. . .	. . .	. . .
EF	73.823768	179.310206	0.41	0.7082

NOTE: The X'X matrix has been found to be singular, and a generalized inverse was used to solve the normal equations. Terms whose estimates are followed by the letter 'B' are not uniquely estimable.

ACT= antifungal activity, D= hardness, B= softness, P=chemical potential, EN= electronegativity, and EF= electrophilicity.

Table S16. Results obtained via multiple regression analysis for series I (**6** and **7a-g**) to *Aspergillus niger* 48 h.

Dependent variable: ACT

Source	DF	Sum of Squares	Mean Square	F Value	Pr > F
Model	1	0.11657693	0.11657693	6.55	0.0507
Error	5	0.08904695	0.01780939		
Corrected Total	6	0.20562389			

R-Square	Adj R-Sq	Coeff Var	Root MSE	ACT Mean
0.786247	0.566943	37.20952	0.133452	0.358650

Source	DF	TYPE III SS	Mean Square	F Value	Pr > F
D	1	0.11657693	0.11657693	6.55	0.0507

Parameter	Estimate	Standar Error	t Value	Pr > |t|
Intercept	-4.573020728	1.92823915	-2.37	0.0638
D	1.252805340	0.48966810	2.56	0.0507

ACT= antifungal activity and D= hardness.

Table S17. Results obtained via multiple regression analysis for series I (**6** and **7a-g**) to *Aspergillus niger* 72 h.

Dependent variable: ACT

Source	DF	Sum of Squares	Mean Square	F Value	Pr > F
Model	1	0.56177610	0.56177610	6.39	0.0648
Error	4	0.35147641	0.08786910		
Corrected Total	5	0.91325251			

R-Square	Adj R-Sq	Coeff Var	Root MSE	ACT Mean
0.92226	0.615138	40.82650	0.296427	0.726066

Source	DF	TYPE III SS	Mean Square	F Value	Pr > F
D	1	0.56177610	0.56177610	6.39	0.0648

Parameter	Estimate	Standar Error	t Value	Pr > |t|
Intercept	-10.88968881	4.59551855	-2.37	0.0769
D	2.93968753	1.16261959	2.53	0.0648

ACT= antifungal activity and D= hardness.

Table S18. Results obtained via multiple regression analysis for series I (**6** and **7a-g**) to *Aspergillus fumigatus* 48 h.

Dependent variable: ACT

**Source**	**DF**	**Sum of Squares**	**Mean Square**	**F Value**	**Pr > F**
Model	2	0.23205010	0.11602505	12.78	0.0108
Error	5	0.04540897	0.00908179		
Corrected Total	7	0.27745907			

**R-Square**	**Adj R-Sq**	**Coeff Var**	**Root MSE**	**ACT Mean**
0.924603	0.836340	28.65704	0.095298	0.332548

**Source**	**DF**	**TYPE III SS**	**Mean Square**	**F Value**	**Pr > F**
D	1	0.21901368	0.21901368	24.12	0.0044
EF	1	0.12784721	0.12784721	14.08	0.0133

**Parameter**	**Estimate**	**Standar Error**	**t Value**	**Pr > |t|**
Intercept	-16.48565247	3.58348397	-4.60	0.0058
D	3.27546195	0.66699526	4.91	0.0044
EF	2.20578882	0.58790088	3.75	0.0133

ACT= antifungal activity, D= hardness, and EF= electrophilicity

Table S19. Results obtained via multiple regression analysis for series I (**6** and **7a-g**) to *Aspergillus fumigatus* 72 h

Dependent variable: ACT

**Source**	**DF**	**Sum of Squares**	**Mean Square**	**F Value**	**Pr > F**
Model	2	0.72462049	0.36231024	12.47	0.0114
Error	5	0.14522701	0.02904540		
Corrected Total	7	0.86984749			

**R-Square**	**Adj R-Sq**	**Coeff Var**	**Root MSE**	**ACT Mean**
0.896573	0.833043	23.34192	0.170427	0.730133

**Source**	**DF**	**TYPE III SS**	**Mean Square**	**F Value**	**Pr > F**
D	1	0.65253793	0.65253793	22.47	0.0052
P	1	0.17862356	0.17862356	6.15	0.0558

**Parameter**	**Estimate**	**Standar Error**	**t Value**	**Pr > |t|**
Intercept	-24.88790861	6.81916671	-3.65	0.0148
D	4.02620570	0.84943817	4.74	0.0052
P	-2.61845192	1.05587846	-2.48	0.055

ACT= antifungal activity, D= hardness, and P=chemical potential.

Table S20. Equations obtained via multiple regression to indexes which statistical analysis was significant and it has two or more important variables to the model for series I.

Microorganism	Time of testing (h)	Equation
C. albicans	24	$MIC = -419.24[\text{mM}] + 53.90\ [\text{mM}\ eV^{-1}]\eta\ [\text{eV}] + 816.92[\text{mM eV}]\ s\ [eV^{-1}]$
C. glabrata	24	$MIC = -675.64[\text{mM}] + 87.50\ [\text{mM}\ eV^{-1}]\eta\ [\text{eV}] + 1306.95[\text{mM eV}]\ s\ [eV^{-1}]$
C. guilliermondii	24	$MIC = -249.19[\text{mM}] + 32.64\ [\text{mM}\ eV^{-1}]\eta\ [\text{eV}] + 477.10[\text{mM eV}]\ s\ [eV^{-1}]$
C. guilliermondii	48	$MIC = -695.84[\text{mM}] + 90.50\ [\text{mM}\ eV^{-1}]\eta\ [\text{eV}] + 1339.019\ [\text{mM eV}]\ s\ [eV^{-1}]$
A. fumigatus	48	$MIC = -16.49[\text{mM}] + 3.28\ [\text{mM}\ eV^{-1}]\eta\ [\text{eV}] + 2.21[\text{mM}\ eV^{-1}]\ \omega\ [\text{eV}]$
A. fumigatus	72	$MIC = -24.89[\text{mM}] + 4.03\ [\text{mM}\ eV^{-1}]\eta\ [\text{eV}] - 2.62[\text{mM}\ eV^{-1}]\ \mu\ [\text{eV}]$

$\boldsymbol{\eta}$= hardness, s= softness, μ=chemical potential, and ω=electrophilicity.
